# Retinal ganglion cells undergo cell type—specific functional changes in a computational model of cone-mediated retinal degeneration

**DOI:** 10.3389/fnins.2023.1147729

**Published:** 2023-05-18

**Authors:** Aiwen Xu, Michael Beyeler

**Affiliations:** ^1^Department of Computer Science, University of California, California, Santa Barbara, CA, United States; ^2^Department of Psychological & Brain Sciences, University of California, California, Santa Barbara, CA, United States

**Keywords:** retina, retinal degeneration, retinal ganglion cells, computational models, retina model

## Abstract

**Introduction:**

Understanding the retina in health and disease is a key issue for neuroscience and neuroengineering applications such as retinal prostheses. During degeneration, the retinal network undergoes complex and multi-stage neuroanatomical alterations, which drastically impact the retinal ganglion cell (RGC) response and are of clinical importance. Here we present a biophysically detailed *in silico* model of the cone pathway in the retina that simulates the network-level response to both light and electrical stimulation.

**Methods:**

The model included 11, 138 cells belonging to nine different cell types (cone photoreceptors, horizontal cells, ON/OFF bipolar cells, ON/OFF amacrine cells, and ON/OFF ganglion cells) confined to a 300 × 300 × 210μm patch of the parafoveal retina. After verifying that the model reproduced seminal findings about the light response of retinal ganglion cells (RGCs), we systematically introduced anatomical and neurophysiological changes (e.g., reduced light sensitivity of photoreceptor, cell death, cell migration) to the network and studied their effect on network activity.

**Results:**

The model was not only able to reproduce common findings about RGC activity in the degenerated retina, such as hyperactivity and increased electrical thresholds, but also offers testable predictions about the underlying neuroanatomical mechanisms.

**Discussion:**

Overall, our findings demonstrate how biophysical changes typified by cone-mediated retinal degeneration may impact retinal responses to light and electrical stimulation. These insights may further our understanding of retinal processing and inform the design of retinal prostheses.

## 1. Introduction

Understanding how the retina responds to light and electrical stimulation is a key issue for neuroscience and neuroengineering applications such as retinal prostheses. Computational models have been built either at the single-cell level or network level to understand the response properties of the healthy retina (for a recent review, see Guo et al., 2014). These include single-compartment models (“point models”) to simulate neuronal response as a function of ionic currents flowing across the neuronal membrane (e.g., Fohlmeister et al., 1990; Fohlmeister and Miller, 1997; Wohrer and Kornprobst, 2009), morphologically realistic models based on detailed anatomical representations of the physical components of biological neurons (e.g., Smith, 1995; Greenberg et al., 1999), and convolutional neural networks (e.g., McIntosh et al., 2016). Several studies did not just focus on the retina's light response but also on the response to electrical stimulation (Cottaris and Elfar, 2005; Guo et al., 2016; Werginz et al., 2018; Beyeler, 2019; Paknahad et al., 2020), which may inform treatment options for people blinded by retinal degenerative diseases.

However, the retinal network undergoes drastic neuroanatomical alterations during retinal degeneration (Marc et al., 2003; Jones et al., 2016) such as retinitis pigmentosa, which are of clinical importance to rehabilitative strategies such as retinal prostheses (Stingl et al., 2013; Luo et al., 2016; Palanker et al., 2020). These alterations are complex and multi-stage (Marc et al., 2003), starting with photoreceptor stress that leads to outer segment truncation (Phase I) and progressive photoreceptor cell death (Phase II), followed by a protracted period of global cell migration and cell death (Phase III). The consequences of these alterations on RGC firing are manifold, which include hyperactivity (Pu et al., 2006; Stasheff, 2008; Stasheff et al., 2011; Telias et al., 2019), emergence of oscillations (Margolis et al., 2014; Ahn et al., 2022), and increased electrical stimulation thresholds (Rizzo et al., 2003b; O'Hearn et al., 2006; Jensen and Rizzo, 2008; Goo et al., 2011; Cho et al., 2016). Oscillations are thought to arise from the network of electrically-coupled AII amacrine cells and ON cone bipolar cell (Haq et al., 2014; Trenholm and Awatramani, 2015; Ahn et al., 2022). Previous research has also identified retinoic acid as a trigger for hyperactivity (Telias et al., 2019).

Previous computational work modeled retinal degeneration, but often stopped short of simulating the global retinal remodeling typified by the progressive nature of these diseases. For instance, Cottaris and Elfar (2005) built a model of the healthy retina and removed the cone population without addressing biophysical changes to the inner retina. Golden et al. (2018) simulated degeneration by removing a fraction of simulated neurons, increasing connectivity among the surviving neurons, and increasing the noise level, but did not address the progressive nature of these diseases. Other models stopped at reducing the thickness of different retinal layers (Paknahad et al., 2020, 2021) or hard-coded known physiological changes, such as increased spontaneous activity, into their model (Loizos et al., 2018). To the best of our knowledge, a comprehensive computational model of retinal degeneration is still lacking.

To address this, we built a biophysically inspired *in silico* computational model of the cone pathway in the retina and simulated the network-level response to both light and electrical stimulation. After verifying that the model reproduced seminal findings about the light response of RGCs, we systematically introduced anatomical and neurophysiological changes to the network and studied their effect on network activity. In early phases of this simulated cone-mediated retinal degeneration, we found that reduced light sensitivity and subsequent death of cones differentially affected ON and OFF RGC firing: whereas the light response of ON RGCs diminished more quickly than that of OFF RGCs, the spontaneous firing rate of OFF RGCs steadily increased. In late phases of degeneration, we found that migration and progressive death of inner retinal neurons led to a steady increase in electrical activation thresholds of both ON and OFF RGCs, especially for epiretinal stimulation.

Our findings demonstrate how biophysical changes associated with cone-mediated retinal degeneration affect retinal responses to both light and electrical stimulation. A detailed model of the retina in health and disease has the potential to further our understanding of visual processing in the retina. It may also inform the design of retinal prostheses, for the effective treatment of inherited retinal degenerative diseases such as retinitis pigmentosa and age-related macular degeneration.

## 2. Methods

**Figure 1:**
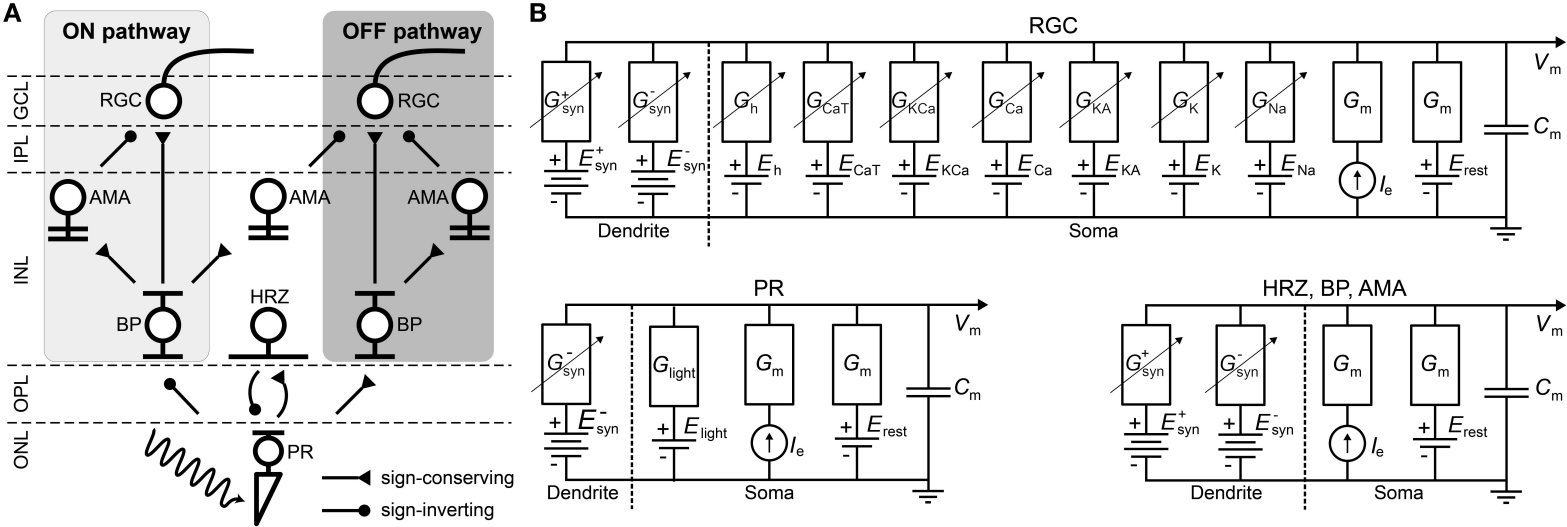
**(A)** Diagram of the connections between the retinal neurons in the healthy state. PR, photoreceptor; HRZ, horizontal cell; BP, bipolar cell; AMA, amacrine cell; RGC, retinal ganglion cell; ONL, outer nuclear layer; OPL, outer plexiform layer; INL, inner nuclear layer; IPL, inner plexiform layer; GCL, ganglion cell layer. **(B)** RC circuit model of a neuron's membrane potential. All neurons included a membrane capacitance (*C*_*m*_), a leakage current (*I*_leak_), an external current driven by the extracellular potential gradient (*I*_*e*_), and synaptically gated ionic currents ($I_{\text{syn}}^{+}$ and $I_{\text{syn}}^{-}$). RGCs had additional voltage-gated and ligand-gated ionic currents (Guo et al., 2016) and photoreceptors had a photo-sensitive current (*I*_light_) as described in Cottaris and Elfar (2005). Dendritic trees and ganglion cell axons were not modeled.

Inspired by Cottaris and Elfar (2005), we started by simulating a three-dimensional healthy patch (300 × 300 × 210 μm) of the cone pathway in the parafoveal retina. The network consisted of 11, 138 cells belonging to nine different cell types (4, 149 cones, 537 horizontal cells, 3, 508 ON/OFF bipolar cells, 779 ON/OFF wide-field amacrine cells, 723 narrow-field amacrine cells, and 1, 442 ganglion cells), connected via generally accepted (Wassle and Boycott, 1991; Rodieck, 1998) synaptic connections (see Figure 1).

Briefly, upon photoactivation, cone photoreceptors (labeled “PR” in Figure 1A) produced a photocurrent that led to hyperpolarization in OFF bipolar cells and depolarization in ON bipolar cells (“BP”). In addition, cones excited horizontal cells (“HRZ”), which in turn inhibited cone terminals, thus generating an inhibitory surround in the bipolar cell response. ON and OFF bipolar cells then excited ON and OFF amacrine cells (“AMA”) as well as ON and OFF ganglion cells (“RGC”), respectively, to generate an inhibitory surround in the ganglion cell response. ON and OFF amacrine cells also provided lateral inhibition to ON and OFF ganglion cells, respectively. Lastly, we included a unilateral inhibitory connection from a special type of narrow-field ON amacrine cell to OFF ganglion cells (Wyatt and Rizzo, 1996). Rod circuitry was not implemented.

The retina model was implemented using Brian 2 (Stimberg et al., 2019) and Brian2GeNN (Stimberg et al., 2020) in Python. All simulations were run on a single NVIDIA RTX 3090 (24 GB of GPU memory), and all our code is available at https://github.com/bionicvisionlab/2023-Xu-Retinal-Degeneration.

### 2.1. Modeling individual neurons

To implement the biophysical properties of retinal neurons, we largely followed Cottaris and Elfar (2005) to modify a leaky integrator model by adding membrane and synaptic conductances (Figure 1B). We assumed that neurons are electronically compact, so their activation levels could be described by a single membrane potential. All 11, 138 neurons had a spatially nonzero soma with non-gated ion channels (leakage channels) modeled by a constant linear conductance (*G*_m_) in series with a constant single-cell battery (i.e., the cell's resting voltage, *E*_rest_) and an extracellular current that modeled extracellular electrical stimulation.

With the exception of RGCs, all other cell types (labeled “HRZ,” “BP,” “AMA” in Figure 1B) were modeled as leaky integrators (Cottaris and Elfar, 2005), whose membrane potential (*v*_m_) followed the following differential equation:


(1) $$\begin{array}{l} C_{\text{m}}\frac{dv_{\text{m}}}{dt}=\underset{\text{syn}}{\sum }i_{\text{syn}}+i_{\text{ext}}+i_{\text{leak}}, \end{array}$$

where *C*_m_ was the cell type–specific membrane conductance, the sum was over all presynaptic currents *i*_syn_ (see Section 2.2), *i*_ext_ was the external current resulting from extracellular electrical stimulation (see Section 2.5), and *i*_leak_ was a leakage current modeled by a constant linear conductance (*G*_m_) in series with a constant single-cell battery (i.e., the cell's resting voltage, *E*_rest_):


(2) $$i_{\text{leak}}=-G_{\text{m}}(v_{\text{m}}-E_{\text{rest}}).$$

It is worth noting that in reality these cell types contain a variety of voltage-gated and ligand-gated ion channels. However, modeling the behavior of these neurons with multiple ion channels would have further increased the complexity and computational cost of the model. For the sake of practical feasibility, we therefore had to limit ourselves to a single ion channel. Cottaris and Elfar (2005) demonstrated that this can still lead to realistic light responses.

The light-cone interaction of photoreceptors (labeled “PR” in Figure 1B) was modeled with an additional current as a synapse, described in detail in Cottaris and Elfar (2005) and given as:


(3) $$\begin{array}{ll} C_{\text{m}}\frac{dv_{\text{m}}}{dt} & =\underset{\text{syn}}{\sum }i_{\text{syn}}+i_{\text{ext}}+i_{\text{leak}}+i_{\text{light}}, \end{array}$$

(4) $$i_{\text{light}}\;=-g_{\text{light}}(v_{\text{m}}-E_{\text{light}})$$

where *E*_light_ = −8mV was the reversal potential and *g*_light_ was the synaptic conductance that depended on the time-dependent light intensity *l*(*t*)∈[0, 1]:


(5) $$g_{\text{light}}=G_{\text{light}}(1-l(t)).$$

As one of our main goals was to study the RGC response to electrical stimulation, which is often delivered by short biphasic pulses, we considered it important that our simulated RGC population exhibited detailed temporal responses. Thus, our implementation of RGCs (labeled “RGC” in Figure 1B) deviated from Cottaris and Elfar (2005), as they were modeled as Hodgkin-Huxley neurons with seven ion channels (Equation 6) that were previously shown to capture the firing dynamics of RGCs in the rabbit retina (Fohlmeister and Miller, 1997; Guo et al., 2016):


(6) $$\begin{array}{l} C_{m}\frac{dV_{\text{m}}}{dt}=\underset{\text{syn}}{\sum }i_{\text{syn}}+i_{\text{ext}}+i_{\text{ion}}, \end{array}$$

where the ionic current *i*_ion_ was given as the product of the neuron's surface area *A* and the sum of several ionic current densities:


(7) $$\begin{array}{ll} i_{\text{ion}} & =A(j_{\text{Na}}+j_{\text{Ca}}+j_{\text{K}}+j_{\text{KA}}+j_{\text{KCa}}+j_{\text{h}}+j_{\text{CaT}}+j_{\text{leak}}),\\ j_{\text{Na}} & =-G_{\text{Na}}m^{3}h(v_{\text{m}}-E_{\text{Na}}),\\ j_{\text{Ca}} & =-G_{\text{Ca}}c^{3}(v_{\text{m}}-E_{\text{Ca}}),\\ j_{\text{K}} & =-G_{\text{K}}n^{4}(v_{\text{m}}-E_{\text{K}}),\\ j_{\text{KA}} & =-G_{\text{KA}}m_{\text{A}}^{3}h_{\text{A}}(v_{\text{m}}-E_{\text{K}}),\\ j_{\text{KCa}} & =-G_{\text{KCa}}m_{\text{KCa}}(v_{\text{m}}-E_{\text{K}}),\\ j_{\text{h}} & =-G_{\text{h}}n_{\text{h}}(v_{\text{m}}-E_{\text{h}}),\\ j_{\text{CaT}} & =-G_{\text{CaT}}m_{\text{T}}^{3}h_{\text{T}}(v_{\text{m}}-E_{\text{Ca}}),\\ j_{\text{leak}} & =-G_{\text{m}}(v_{\text{m}}-E_{\text{rest}}). \end{array}$$

Here, *j*_Na_ was a voltage-gated sodium channel with gating variables *m* and *h*; *j*_Ca_ was a voltage-gated calcium channel with gating variable *c* and *E*_Ca_ modeled with the Nernst equation (see Guo et al., 2016 for details); *j*_K_ was a non-inactivating potassium channel with gating variable *n*; *j*_KA_ was an inactivating potassium channel with gating variables *m*_A_ (called *A* in Guo et al., 2016) and *h*_A_; *j*_KCa_ was a Ca^2+^-activated potassium channel gated by *m*_KCa_ whose value was dependent on the internal calcium concentration (see Guo et al., 2016 for details); *j*_h_ was a hyperpolarization-activated non-selective cationic channel with gating variable *n*_h_; and *j*_CaT_ was a low-threshold voltage-activated calcium channel with gating variables *m*_T_ and *h*_T_ (Fohlmeister and Miller, 1997; Guo et al., 2016). All neuron parameters are given in Table 1.

**Table 1:** Neuronal membrane parameters.

	**PR**	**HRZ**	**BP_ON/OFF_**	** ${\text{AMA}}_{\text{ON/OFF}}^{\text{WF/NF}}$ **	**RGC_ON_**	**RGC_OFF_**
*C*_m_ ( pF )	80.0	210.0	50.0	50.0	50.0	50.0
*G*_m_ ( nS )	4.0	2.5	2.0	2.0	—	—
*G*_m_ ( mS cm^−2^ )	—	—	—	—	0.3	0.274
*G*_light_ ( nS )	0.9	—	—	—	—	—
*E*_rest_(*mV*)	−50.0	−65.0	−45.0	−50.0	−66.5	−70.5
*E*_Na_(*mV*)	—	—	—	—	35.0	35.0
*E*_K_(*mV*)	—	—	—	—	−72.0	−68.0
*E*_h_(*mV*)	—	—	—	—	−45.8	−26.8
*G*_Na_ ( mS cm^−2^ )	—	—	—	—	1072.0	249.0
*G*_K_ ( mS cm^−2^ )	—	—	—	—	40.5	68.85
*G*_KA_ ( mS cm^−2^ )	—	—	—	—	94.5	18.9
*G*_Ca_ ( mS cm^−2^ )	—	—	—	—	2.1	1.6
*G*_KCa_ ( mS cm^−2^ )	—	—	—	—	0.04	0.0474
*G*_h_ ( mS cm^−2^ )	—	—	—	—	0.4287	0.1429
*G*_CaT_ ( mS cm^−2^ )	—	—	—	—	0.008	0.1983

*G*_m_ for RGCs was set such that the spontaneous firing rate was around 2 Hz. All other values for *G*_m_ and *C*_m_ were adopted from Cottaris and Elfar (2005). All others were adopted from Guo et al. (2016).

The equations for the gating variables were identical to Guo et al. (2016) (see their Tables 2, 3). In short, all gating variables, except the inactivating gating variable *h*_*T*_ of *j*_*CaT*_, followed first-order kinetics:


(8) $$\begin{array}{l} \frac{dx}{dt}=\alpha_{x}\left(1-x\right)-\beta_{x}x, \end{array}$$

where *x* was the gating variable, α_*x*_ was the opening rate and β_*x*_ was the closing rate of the channel. The inactivating gating variable *h*_T_ of *j*_CaT_ (but not *m*_T_; note the typo in Guo et al., 2016) followed second-order dynamics:


(9) $$\begin{array}{ll} \frac{dh_{\text{T}}}{dt} & =\alpha_{h_{\text{T}}}\left(1-h_{\text{T}}-d_{\text{T}}\right)-\beta_{h_{\text{T}}}h_{\text{T}}, \end{array}$$

(10) $$\begin{array}{ll} \frac{d\left(d_{\text{T}}\right)}{dt} & =\alpha_{d_{\text{T}}}\left(1-h_{\text{T}}-d_{\text{T}}\right)-\beta_{d_{\text{T}}}d_{\text{T}}. \end{array}$$

The initial values of the gating variables and the internal calcium concentrations of the RGCs can be found in Appendix A.4.

**Table 2:** Spatial layout parameters for the different cell types (Cottaris and Elfar, 2005).

	**PR**	**HRZ**	**BP_ON/OFF_**	** ${\text{AMA}}_{\text{ON/OFF}}^{\text{WF}}$ **	** ${\text{AMA}}_{\text{ON}}^{\text{NF}}$ **	**RGC_ON/OFF_**
λ ( μm )	2.5	7.0	3.85	8.0	6.0	6.0
*z*_min_ ( μm )	170	100	100	80	80	25
*z*_max_ ( μm )	205	128	128	101	101	39
*G*_ext_ ( nS )	4.0	2.5	2.0	2.0	2.0	2.0

PR, photoreceptor; HRZ, horizontal cell; BP, bipolar cell; AMA, amacrine cell; RGC, retinal ganglion cell; WF, widefield; NF, nonwide-field.

**Table 3:** Synaptic parameters.

	**τ ( ms )**	***E*_syn_ ( mV )**	***G*_min_ ( nS )**	***G*_max_ ( nS )**	***V*_50_ ( mV )**	**β ( mV )**	**Type**	**σ ( μm )**
PR → HRZ	7	0.0	0.0	7.0	−43.0	2.0	I	10.5
HRZ → PR	7	−67.0	0.0	3.0	−29.5	7.4	I	2.5
PR → BP_ON_	5	0.0	0.1	1.1	−47.0	1.7	D	3.85
PR → BP_OFF_	13	0.0	0.0	3.75	−41.5	1.2	I	3.85
${\text{BP}}_{\text{ON}}\to {\text{AMA}}_{\text{ON}}^{\text{WF}}$	5	0.0	0.0	1.0	−33.5	3.0	I	24.0
${\text{BP}}_{\text{ON}}\to {\text{AMA}}_{\text{ON}}^{\text{NF}}$	5	0.0	0.0	0.2	−35.0	3.0	I	6.0
${\text{BP}}_{\text{OFF}}\to {\text{AMA}}_{\text{OFF}}^{\text{WF}}$	11	0.0	0.0	1.8	-44.0	3.0	I	24.0
BP_ON_ → RGC_ON_	5	0.0	0.0	2.5	−33.5	3.0	I	6.0
${\text{AMA}}_{\text{ON}}^{\text{WF}}\to {\text{RGC}}_{\text{ON}}$	5	−70.0	0.0	2.0	−42.5	2.5	I	6.0
BP_OFF_ → RGC_OFF_	13	0.0	0.0	2.5	−44.0	3.0	I	6.0
${\text{AMA}}_{\text{OFF}}^{\text{WF}}\to {\text{RGC}}_{\text{OFF}}$	12	−70.0	0.0	2.5	−34.4	2.5	I	6.0
${\text{AMA}}_{\text{ON}}^{\text{NF}}\to {\text{RGC}}_{\text{OFF}}$	12	−80.0	0.0	2.0	−47.5	2.0	I	6.0

The synaptic delays were set such that the latency of ON RGCs were around 20 ms and the latency of OFF RGCs were around 50 ms. All other values are from Cottaris and Elfar (2005).

Neurons were assumed to contain a spherical soma with either 26 μm diameter in the case of RGCs (Crooks and Kolb, 1992; Milo et al., 2010) or 7 μm otherwise (Cottaris and Elfar, 2005). The initial values of the membrane voltages were set according to normal distributions, whose parameters can be found in Appendix A.4.

Ionic current densities were multiplied by the surface area *A* of the RGC to convert to a current. *G*_leak_ was set to a value so that the spontaneous firing rate under 0.5 light was around 2 Hz (Tao et al., 2020). A spike was recorded whenever the membrane potential exceeded −10 mV.

### 2.2. Modeling the retinal circuitry

Neurons were connected as shown in Figure 1 using parameters given in Table 3. All neurons belonging to a particular cell type were arranged in a hexagonal mosaic, where the *x* and *y* coordinates of a neuron were given as:


(11) $$\begin{array}{l} \left[\begin{array}{c} x\\ y \end{array}\right]=\left[\begin{array}{cc} i & j \end{array}\right]\times \left[\begin{array}{cc} 1 & \sqrt{3}\\ 1 & -\sqrt{3} \end{array}\right]\lambda , \end{array}$$

where *i, j* = 0, ±1, ±2..., and λ was cell-type specific (Table 2). The *x* and *y* coordinates were further Neurons were confined to different *z* locations depending on their cell type (Table 2). Within each band, the *z* coordinate was assigned by sampling from a random uniform distribution.

Synapses were assumed to lie at the center of a neuron's dendritic field (excitatory if *E*_syn_>*E*_rest_ and inhibitory if *E*_syn_<*E*_rest_). The synaptic connection from presynaptic neurons of the same type to a postsynaptic neuron was modeled with a current *i*_syn_ (see Equations 1, 3, and 6) in the postsynaptic neuron via:


(12) $$\begin{array}{l} i_{\text{syn}}=-g_{\text{syn}}\left(v_{\text{m}}-E_{\text{syn}}\right), \end{array}$$

where *E*_syn_ was different for each synaptic type (see Table 3). *g*_syn_ was computed as a spatially weighted sum over the conductances of all the channels from the presynaptic neurons:


(13) $$\begin{array}{ll} g_{\text{syn}} & =\frac{1}{W}\sum_{p}g_{\text{syn},p}\exp(-\frac{D(p_{0},p)}{\sigma }),\\ W & =\sum_{p}\exp(-\frac{D(p_{0},p)}{\sigma }), \end{array}$$

where *D*(**p**_0_, **p**) was the Euclidean distance between the center of the postsynaptic neuron **p**_0_ and the center of a presynaptic neuron **p**, and σ was a decay constant that determined how the presynaptic currents were weighted. σ was different for each synaptic type (see Table 3).

Following Cottaris and Elfar (2005), we modeled the individual channel conductance *g*_syn, **p**_ as either a monotonically increasing (type “I”) or monotonically decreasing (type “D”) function of the membrane potential of the presynaptic neuron with the following equation:


(14) $$g_{\text{syn}}(t)=\{\begin{array}{ll} G_{\min}+(G_{\max}-G_{\min}) & \\ \times (1-{\left(1+\exp(\frac{v_{\text{pre}}(t-\tau )-V_{50}}{\beta })\right)}^{-1}) & \text{iftypeis “I”}\\ G_{\min}+(G_{\max}-G_{\min}) & \\ \times {\left(1+\exp(\frac{v_{\text{pre}}(t-\tau )-V_{50}}{\beta })\right)}^{-1} & \text{if type is “D”}, \end{array}$$

where *G*_min_ and *G*_max_ were the lower and upper bound of values for the synaptic conductance, *v*_pre_ was the membrane potential of the presynaptic neuron, τ was the synaptic delay, *V*_50_ determined the function's center operating point and β determined the function's steepness (see Table 3).

Synaptic delays (τ in Table 3) were set such that ON RGCs fired their first spikes roughly 20 ms after stimulus onset and OFF RGCs fired roughly 50 ms after stimulus onset (Zeck et al., 2011). To achieve these response times, we assumed a constant transmission speed and calculated the average distance between each connected pair of cells to set τ accordingly.

### 2.3. Modeling retinal degeneration

Inherited retinal degenerative diseases, specifically photoreceptor-initiated ones, are commonly described in the literature as progressing in three phases (Marc et al., 2003; Jones et al., 2016):

- Phase I starts with either cone or rod stress, which leads to the truncation of the outer segments. The population of the affected photoreceptors starts to decrease and their neurites start to extend.
- In Phase II, the other class of photoreceptors also start to die and extend their neurite. Cones continue to truncate. Muller cells move to the outer nuclear layer and start to seal off the retina from the choroid. This process is called subretinal fibrosis, which will later evolve to a glial seal. Horizontal cells begin to hypertrophy and extend their neurites, while rod and cone bipolar cells retract their dendrites.
- Phase III is a protracted period of cell death and leads to global retinal remodeling. In early Phase III, Muller cells hypertrophy and form the glial seal. Neurons start to die, while microneuromas start to form. They often contain active synapses despite lacking normal signaling abilities. In middle Phase III, progressive neuronal death and microneuroma formation continue, while the remaining neurons start to migrate. Specifically, amacrine and bipolar cells move to the inner plexiform and the ganglion cell layer, and amacrine cells and RGCs move to the glial seal. In late Phase III, the microneuromas regress with continued cell death, accompanied with the hypertrophy of Muller cells and vessels.

To make the modeling of such a complex process feasible, we limited ourselves to the major neuroanatomical changes that may have an impact on RGC signaling, and introduced them in a systematic step-wise manner. These changes are summarized in Table 4.

**Table 4:** Phases of retinal degeneration (Marc et al., 2003), with corresponding changes in the retina and in our model.

	**Changes in the retina**	**Changes in the model**
Phase I/II	Cone truncation	Gradual decrease of *G*_light_ (Equation 5)
	Cone cell death	Gradual decrease of cone population
Phase III	Neuronal cell death	Retain 0 % cones and horizontal cells
		Gradual decrease of the population of bipolar and amacrine cells
	Retinal remodeling	Migration of bipolar, amacrine, ganglion cells

#### 2.3.1. Phase I/II

Because our model retina was intended to simulate the parafoveal retinal region and thus did not include rods, we combined Phases I and II into Phase I/II, where we gradually reduced the cone population and shortened the outer segments of the surviving cones, the latter of which was modeled by a gradual reduction in the ceiling of a cone's light response, *G*_light_ (Equation 5). To model disease progression over time (**Figure 4E**), we assumed a linear reduction in cone segment length and cone population over time.

In inherited retinal degeneration, most cones die by the end of Phase I/II (Jones et al., 2016), and the few that remain lose the ability to communicate with the inner retina. Muller cells play an important role in this phase of retinal degeneration, as they tend to move around and start to seal off the retina from the choroid. Although we did not explicitly model the movement and hyperproliferation of Muller cells, we modeled their functional consequence on the cones, which is to isolate them from the other retinal cells. To model this combined effect, we removed all photoreceptors and horizontal cells, which marked the end of Phase I/II.

#### 2.3.2. Phase III

To simulate global retinal remodeling in Phase III, we restricted ourselves to simulating cell death and migration.

First, we gradually reduced the population of bipolar and amacrine cells (but not RGCs). Second, according to the literature, a fraction of the surviving amacrine and bipolar cells tend to migrate to the inner plexiform layer and the ganglion cell layer, whereas amacrine cells and RGCs tend to migrate to the glial seal (Marc et al., 2003). We simulated this by migrating a randomly chosen subset of cells to different layers. A subset of amacrine cells was moved to the horizontal cell layer (*z*∈[100, 128μm], close to the hypothetical glial seal), the inner plexiform layer (*z*∈[40, 80μm]), and the ganglion cell layer (*z*∈[25, 39μm]) in equal proportions. Half of the migrating bipolar cells were moved to the inner plexiform layer and the other half to the ganglion cell layer. The migrating RGCs were moved to the horizontal cell layer. The *z* coordinates of the migrating cells were sampled from a random uniform distribution in the respective range of *z* values that make up the different retinal layers (Table 5), whereas *x*, *y* coordinates remained unchanged. Synaptic weights and delays were unaffected by these coordinate changes.

**Table 5:** The range of *z* coordinates (*z*_min_, *z*_max_) where bipolars, amacrines, and RGCs could be found during degeneration, given in μm.

	**BP_ON/OFF_**	** ${\text{AMA}}_{\text{ON/OFF}}^{\text{WF/NF}}$ **	**RGC_ON/OFF_**
Healthy (*z*_min_, *z*_max_)	(100, 128)	(80, 101)	(25, 39)
Degenerated (*z*_min_, *z*_max_)	(25, 39)	(25, 39)	(100, 128)
	(40, 80)	(40, 80)	–
	–	(100, 128)	–

The bipolar cells migrated to the inner plexiform layer and the ganglion cell layer. The amacrine cells migrated to the horizontal cell layer, the inner plexiform layer and the ganglion cell layer. The RGCs migrated to the horizontal cell layer.

To model disease progression over time (**Figure 4E**), we assumed a linear reduction in cell survival rate (from 100 % at the beginning of Phase III to 0 at the end of Phase III) and a linear increase in cell migration rate (from 0 to 50 %).

Our model did not include Muller cells or microneuromas. However, some of the modeled changes may be indirectly due to Muller cell activity, such as the progressive death of inner retinal neurons.

### 2.4. Estimating spatiotemporal receptive fields

To measure the spatiotemporal receptive field of an RGC, we fit a generalized linear model to its spiking response to a spatially correlated “cloud” stimulus (Shi et al., 2019). The stimulus consisted of spatiotemporal Gaussian white noise (pixel size = 6.25μm, mean brightness = 0.5, standard deviation of brightness = 0.175, refresh rate = 20 Hz) filtered with a two-dimensional spatial Gaussian filter (standard deviation = 12.5μm). The resulting spikes were binned at the refresh rate of the stimulus.

The generalized linear model predicted the firing rate of a RGC as:


(15) $$r(t)=f(\sum_{i}k_{i}s_{i}(t)),$$

where *s*_*i*_(*t*) denoted the relevant stimulus frames before and at time *t*, *k*_*i*_ denoted the spatial filter for the stimulus frame *s*_*i*_(*t*), and *f* was a nonlinear function. We trained a generalized linear model in PyTorch with a spatiotemporal filter spanning five time steps and a rectified linear unit (ReLU) as the nonlinear function. The generalized linear model was regularized with a Laplacian square penalty on the spatial filters (Shi et al., 2019). We used the mean squared error as loss function. The generalized linear model was trained for 3,000 epochs with the Adam optimizer and a learning rate of 0.000001 decayed by 10 % every 2,000 epochs.

### 2.5. Modeling extracellular electrical stimulation

Extracellular electrical stimulation was assumed to be generated by a disk electrode with diameter αμm placed at (*x*_*e*_, *y*_*e*_, *z*_*e*_). The extracellular electrical potential *v*_ext_ at location (*x, y, z*) was given by:


(16) $$v_{\text{e}}(x,y,z)=\frac{2V_{0}}{\pi }\arcsin(\frac{2\alpha }{\sqrt{{\left(r+\alpha \right)}^{2}+d^{2}}+\sqrt{{\left(r-\alpha \right)}^{2}+d^{2}}}),$$

where *V*_0_ was the electrical potential of the disk, $r=\sqrt{{\left(x-x_{e}\right)}^{2}+{\left(y-y_{e}\right)}^{2}}$, and *d* = *z*−*z*_*e*_ (Wiley and Webster, 1982). *v*_e_ was converted to a current, *i*_e_ (see Equations 1, 3, and 6), as follows:


(17) $$\begin{array}{l} i_{\text{e}}=\frac{1}{2}G_{\text{ext}}\langle v_{\text{e}}\rangle , \end{array}$$

where *G*_ext_ was a conductance (see Table 2) and 〈*v*_*e*_〉 was the average of the absolute voltage differences between 500 uniformly sampled, diametrically opposing points on the neuron's spherical soma (Knuth, 1969).

Although epiretinal electrodes are known to activate passing axon fibers (Rizzo et al., 2003a; Fried et al., 2009; Beyeler et al., 2019), we did not include RGC axons in our simulations.

The epiretinal electrode was centered at (*x, y, z*) = (0, 0, −2μm). The subretinal electrode was centered at (*x, y, z*) = (0, 0, 135μm). Both electrodes had a diameter of α = 80μm.

## 3. Results

### 3.1. Light response of the healthy retina

**Figure 2:**
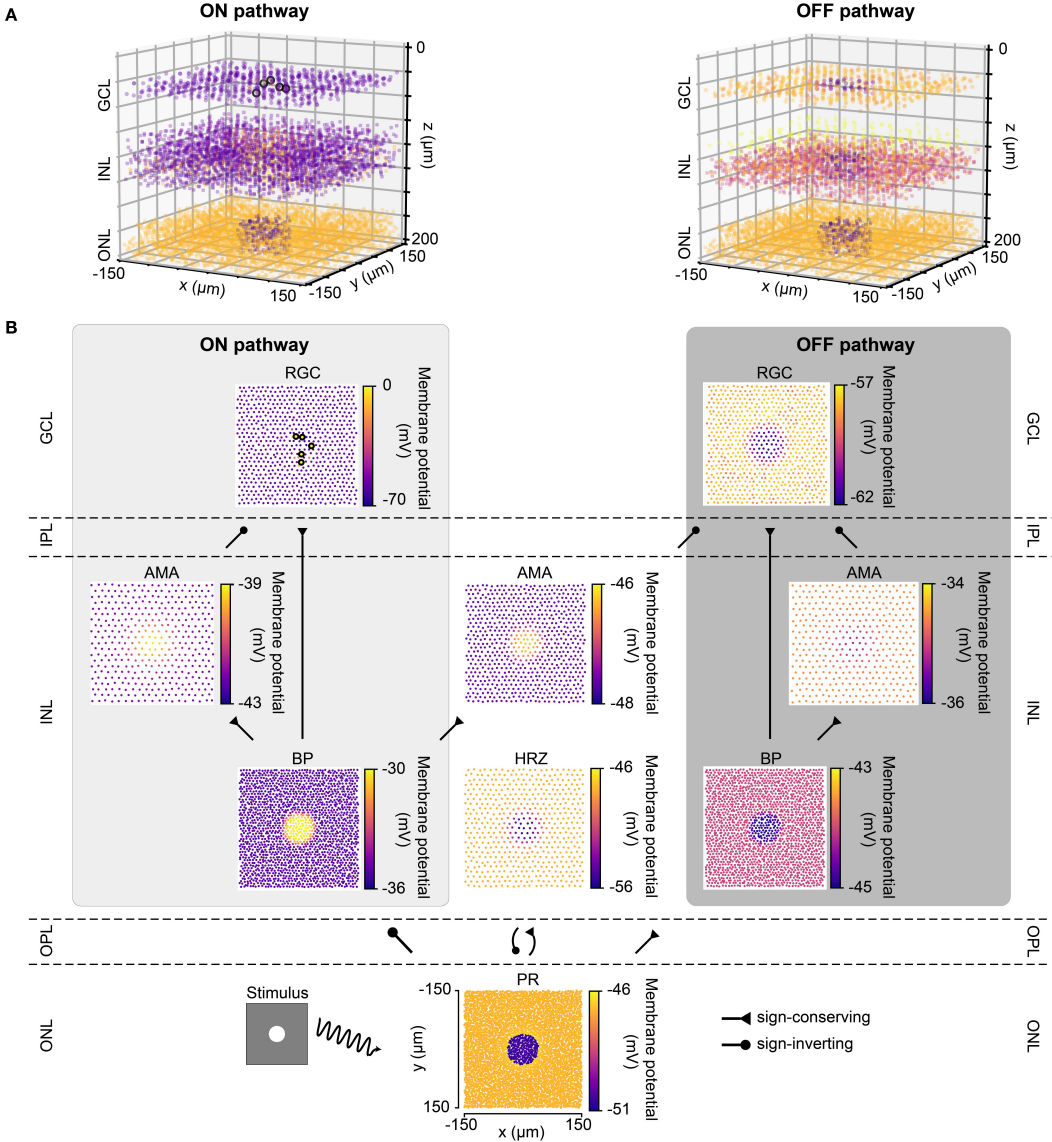
The light response of the retinal network in the healthy state, presented both in 3D and by cell type. Abbreviations same as in Figure 1. **(A)** The light response of the healthy retina presented separately for the ON pathway (left) and the OFF pathway (right) in 3D. The light stimulus that was used to elicit the response was a bright disk (40 μm in radius with light intensity 1) placed at the center of a gray background (300 × 300 μm with light intensity 0.5; illustrated by the bottom left inset of *B*). The light response shown occurred 110 ms after stimulus onset. Each circle represents the (*x, y, z*) location of a neuron, and the color of each circle indicates the membrane potential of each neuron, with the color bars in *B*. Enlarged circles with black border indicate neuronal spikes. The plot of the ON pathway includes cone photoreceptors, horizontal cells, and all the cells belonging to the ON pathway, and the plot of the OFF pathway includes cone photoreceptors, horizontal cells, and all the cells belonging to the OFF pathway. **(B)** The light response presented by cell type, corresponding to each layer in *A*. Each circle represents the x-y location of a neuron, and the color of each circle indicates the membrane potential of each neuron. Enlarged circles with black border indicate neuronal spikes. For an animated version of this figure, see Supplementary Video 1.

Figure 2 shows the response of the healthy retina to a bright disk stimulus (40μm in radius, with light intensity 1.0) presented against a gray background (300 × 300μm, with light intensity 0.5). Figure 2B breaks down the retinal response to the disk stimulus layer by layer. Upon stimulus onset, photoreceptors in the center of the mosaic became hyperpolarized and were surrounded by a thin ring of depolarized cells due to reduced lateral inhibition provided by the horizontal cells. This activity spread through both ON and OFF pathways, leading to depolarized ON bipolar cells and spiking ON RGCs, whereas corresponding cells in the OFF pathway were hyperpolarized.

**Figure 3:**
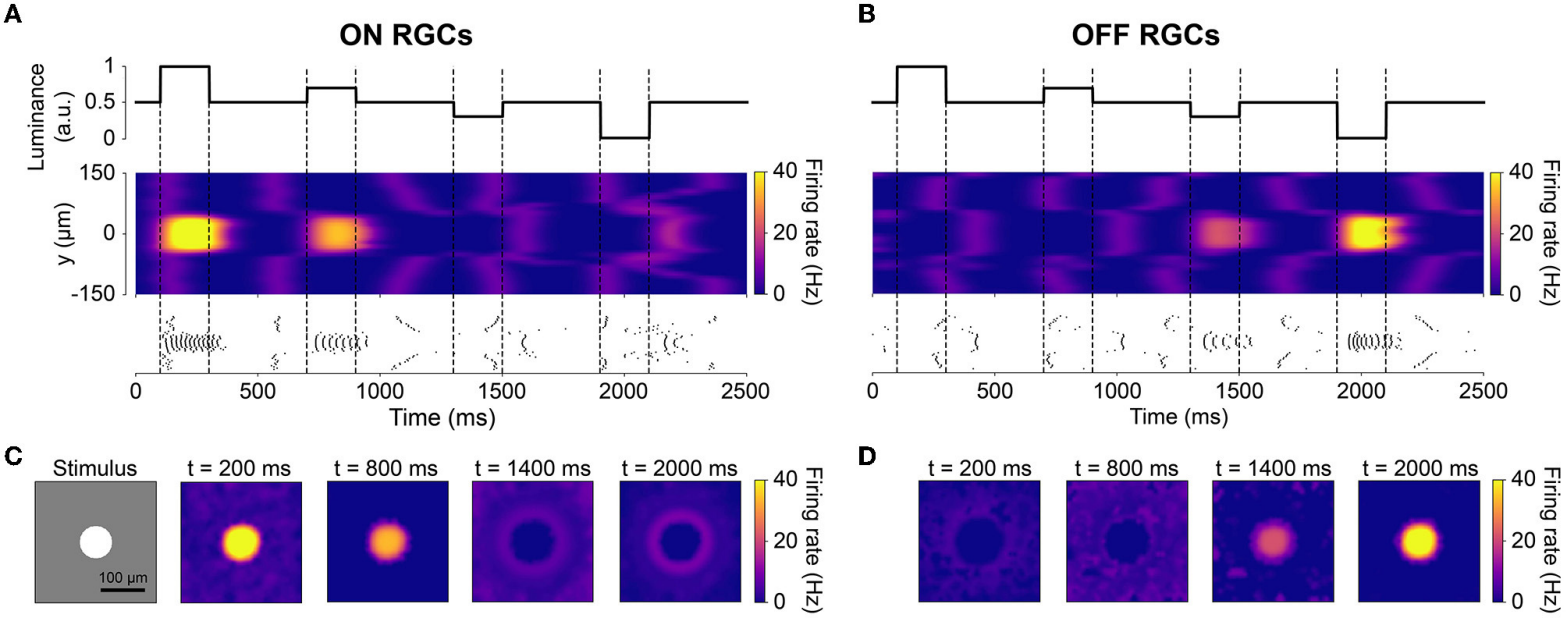
The spatiotemporal response of RGCs to a temporally varying light stimulus (inspired by Cottaris and Elfar, 2005). The light stimulus (illustrated in the bottom-left inset) was a disk (40 μm in radius) placed at the center of a gray background (300 ×300μm with light intensity 0.5), varying in intensity over time. **(A, B)** Spatiotemporal profile of RGC firing rate for neurons located at *x* = 0μm, visualized both as a heatmap (smoothed with a 50 ms Gaussian sliding window) and raster plot. The vertical dotted lines indicate the time of change in light intensity of the disk stimulus. **(C, D)** Spatial activity profiles of RGC firing rate taken at different points in time.

The spatiotemporal evolution of RGC activity in response to the above mentioned stimulus is shown in Figure 3, which was modeled after Figure 5 in Cottaris and Elfar (2005). The stimulus mentioned above was modulated in time by a square wave signal (200 ms phase duration) at four contrast levels: +100, +50, −50, −100 %. Consistent with conduction delays in the rabbit retina (Zeck et al., 2011), ON RGCs first fired roughly 20 ms after stimulus onset, whereas OFF RGCs took 50 ms to respond (Figures 3A, B). RGC unaffected by the stimulus exhibited at a 2 Hz spontaneous firing rate, which was achieved by setting the conductance of the leakage current (see Section 2.1). Synchronization of firing increased with stimulus strength for both ON and OFF populations. The center-surround structure of RGC receptive fields is evident in Figures 3C, D.

### 3.2. Retinal degeneration differentially affects the spontaneous firing of ON and OFF cells

**Figure 4:**
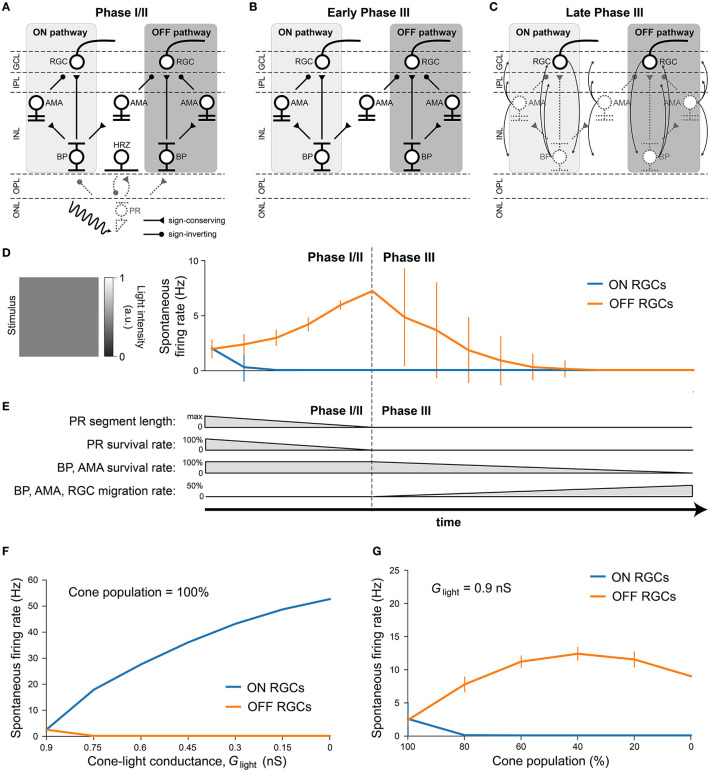
Simulating retinal degeneration. Abbreviations same as in Figure 1. **(A)** To simulate Phase I/II of retinal degeneration, we gradually shortened the cone outer segment length while simultaneously reducing the cone population. **(B)** The complete loss of the cone population marks the beginning of Phase III. **(C)** During Phase III, we gradually reduced the population of bipolar and amacrine cells. In addition, a fraction of surviving cells migrated to different layers: amacrine cells started to migrate to the horizontal cell layer, the inner plexiform layer, and the ganglion cell layer; bipolar cells started to migrate to the inner plexiform layer and the ganglion cell layer; RGCs started to migrate to the horizontal cell layer. At the end of Phase III, all inner retinal neurons have degenerated. **(D)** Spontaneous firing rate of ON and OFF RGCs as a function of disease progression. Input was a full-field stimulus of *L*(*t*) = 0.5 light intensity (Equation 5). Values indicate the spontaneous firing rate measured over 2,500 ms and averaged across all RGCs, with vertical bars indicating the standard deviation. **(E)** To simulate disease progression over time, we assumed a constant rate of change for PR segment length and neuron survival. **(F)** RGC spontaneous firing rate as a function of cone outer segment truncation (simulated as a reduction in the cone-light conductance *G*_light_, see Equation 5), averaged across RGCs, while the cone population size was held constant. **(G)** RGC spontaneous firing rate as a function of the size of the cone population, averaged across the population of surviving RGCs, while *G*_light_ was held constant.

After verifying the light response of the healthy retina model, we gradually introduced neuroanatomical and neurophysiological changes to the network in order to model retinal degeneration (Figure 4). Retinal degenerative diseases are commonly described in the literature as progressing in three phases (Marc et al., 2003; Jones et al., 2016), starting with photoreceptor stress that leads to outer segment truncation (Phase I) and progressive photoreceptor cell death (Phase II), followed by a protracted period of global cell migration and cell death (Phase III). To make the modeling of such a complex process feasible, we limited ourselves to the major changes that may have an impact on RGC signaling as outlined below (see Section 2.3 for details). Because our model did not include rods, we combined Phases I and II into Phase I/II, where we gradually reduced the cone population and shortened the outer segments of the surviving cones (Figure 4A). The complete loss of photoreceptors and horizontal cells marked the end of Phase I/II (Figure 4B). To simulate global retinal remodeling in Phase III, we gradually reduced the population of bipolar and amacrine cells while migrating a randomly chosen fraction of inner retinal neurons to different retinal layers (Figure 4C). To model disease progression over time (Figure 4E), we assumed a linear reduction in cone segment length and cone population during Phase I/II, and a linear reduction in cell survival rate as well as a linear increase in cell migration rate during Phase III.

Although we did not alter the inherent excitability of RGCs, the network-level changes described above had a profound impact on RGC activity. Figure 4D shows the spontaneous firing rate of RGCs as a function of disease progression, measured over 2500 milliseconds and averaged across all RGCs. Whereas, most ON RGCs were silenced as soon as the cone population and outer segment length dropped below 80 % of their initial values, OFF RGCs experienced a gradual increase in spontaneous firing rate throughout Phase I/II, which peaked at roughly 300 % of its initial value at the beginning of Phase III. After that, the average spontaneous firing rate of the OFF RGC population gradually dropped to zero, although individual cells differed greatly in their activation profiles. Despite the increased variability in mean activity, no bursting or oscillatory activity emerged, as evidenced by Poissonian inter-spike interval distributions (see Figure A1). This could be attributed to the fact that our model did not incorporate direct electrical coupling between cells, which is believed to be responsible for the development of oscillatory behavior in retinal degeneration (Haq et al., 2014; Trenholm and Awatramani, 2015; Ahn et al., 2022).

We identified the underlying mechanistic causes of the change in spontaneous firing rate and found that they differed for ON and OFF RGCs. Whereas, ON cells increased their spontaneous firing mainly as a function of cone outer segment truncation (Figure 4F), OFF cells were mainly affected by the size of the surviving cone population (Figure 4G). The full range of light responses as a function of outer segment truncation and cone population size is given in Figure A2.

### 3.3. Light response of ON cells decreases more quickly than that of OFF cells during degeneration

**Figure 5:**
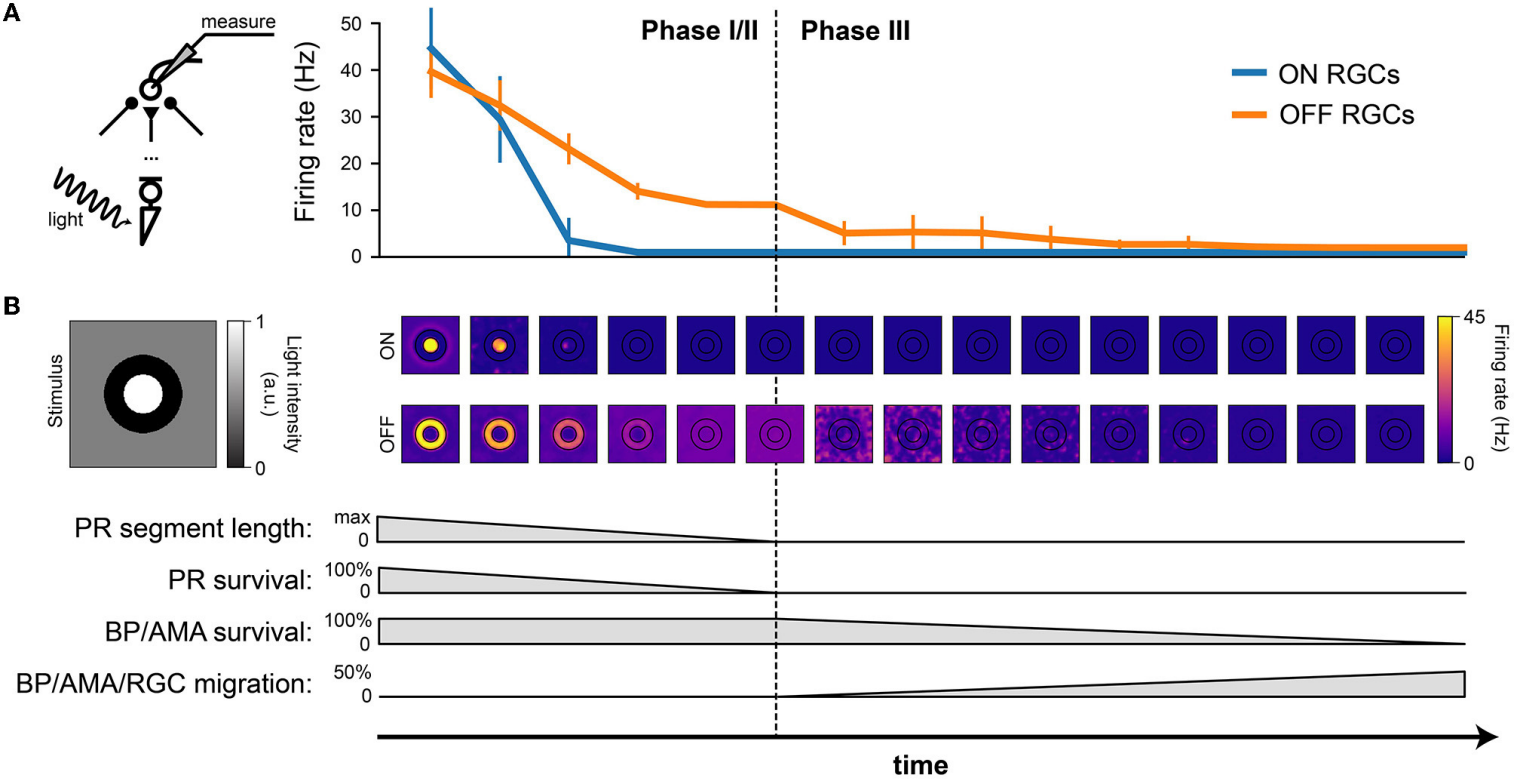
RGC firing rate (in response to light stimulation) decreases with the progression of degeneration. The stimulus was a bright disk (40 μm in radius with light intensity 1) surrounded by a dark ring (40 μm in inner radius and 80 μm in outer radius with light intensity 0) placed on a gray background (300 × 300 μm with light intensity 0.5). **(A)** Firing rate of ON and OFF RGCs, measured over the 1000 ms stimulus presentation, plotted against time. The mean and standard deviation were calculated from the ON RGCs under the bright disk and the OFF RGCs under the dark ring. **(B)** The spatial activity of ON RGCs (top row) and OFF RGCs (bottom row) plotted at different time points of degeneration. The borders of the bright center and dark surround are outlined in black.

To investigate how the light response of RGCs changed as a function of disease progression, we presented a constant stimulus in all stages of the disease (Figure 5). The stimulus was a bright disk (40 μm in radius with maximal light intensity, *l*(*t*) = 1, Equation 5) surrounded by a dark ring (40 μm in inner radius and 80 μm in outer radius with light intensity 0) placed on a gray background simulated with light intensity 0.5 (Figure 5B, *left*). The stimulus was presented for 1,000 ms, during which the mean firing rate of each RGC was calculated.

Whereas, both ON and OFF cells initially responded with similar firing rates, ON cells saw a much quicker reduction in firing rate during Phase I/II than OFF cells, remaining silent for the second half of Phase I/II and all throughout Phase III (Figure 5A).

The spatial response profile at different time steps (where the center of the image is aligned with the corresponding time of disease progression on the *x* axis) is shown Figure 5B. Whereas, the center-surround structure of the retinal response is preserved during early stages of Phase I/II, spatial specificity is quickly lost during later stages of Phase I/II. During Phase III, it is not uncommon for the most active OFF cells to be found far away from the site of stimulation.

### 3.4. Ganglion cells quickly lose spatial selectivity during Phase I/II

To further illustrate the change in spatiotemporal receptive field profiles, we fit a generalized linear model to the spiking response of RGCs to a “cloud” stimulus consisting of spatiotemporal Gaussian white noise filtered with a two-dimensional spatial Gaussian filter (Shi et al., 2019; for details see Section 2.4).

**Figure 6:**
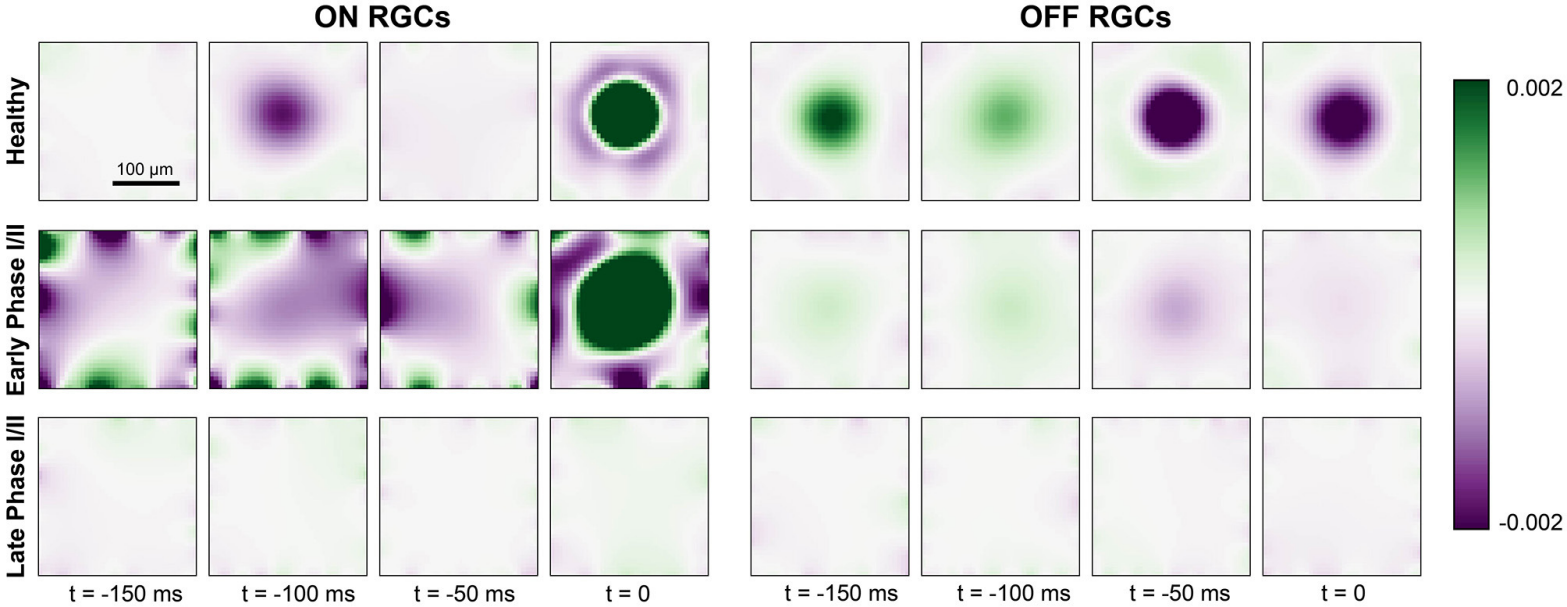
Generalized linear model fit for ON cells (left) and OFF cells (right) for a healthy retina (*top row*), early Phase I/II (*middle row*; 100 % cones surviving and *G*_light_ = 0.75 for ON, 60 % cones surviving and *G*_light_ = 0.6 for OFF), and late Phase I/II (*bottom row*; 80 % cones surviving and *G*_light_ = 0.75 for ON, 40 % cones surviving and *G*_light_ = 0.45). The colorbar represents the range of values of the linear filters *k*_*i*_ (see Section 2.4) at each spatial and temporal location, where green indicates excitatory values and purple indicates inhibitory values.

The fitted spatiotemporal receptive field of two example cells located at the center of the simulated retinal patch is shown in Figure 6. As expected, the receptive field profile of the healthy ON cell showed a clear excitatory center and an inhibitory surround at the time of a spike (*t* = 0ms), with reversed polarity at *t* = −100ms. In early Phase I/II (where cone population and outer segment length were at 80 % of their healthy values), inhibitory subregions at times *t* < 0 were much broader and much more pronounced, and were followed by an enlarged excitatory center that had lost its circular shape. In later stages of Phase I/II (where cone population and outer segment length were at 40 % of their healthy values), the ON cell was no longer sufficiently responsive to light stimulation and its spatial response profile was lost.

The receptive field profile of the OFF cell also exhibited a center-surround structure, though the excitatory surround was most pronounced at *t* = −50ms due to the longer synaptic delay of the OFF pathway (see Section 2.2). This also led to a prolonged response at the center of the receptive field profile. In early Phase I/II, the spatial response profile of the OFF cell seemed to be largely preserved, although the overall response as weakened. In late Phase I/II, the OFF cell was no longer sufficiently responsive to light stimulation and its spatial response profile was lost.

### 3.5. Electrical thresholds increase throughout retinal degeneration

**Figure 7:**
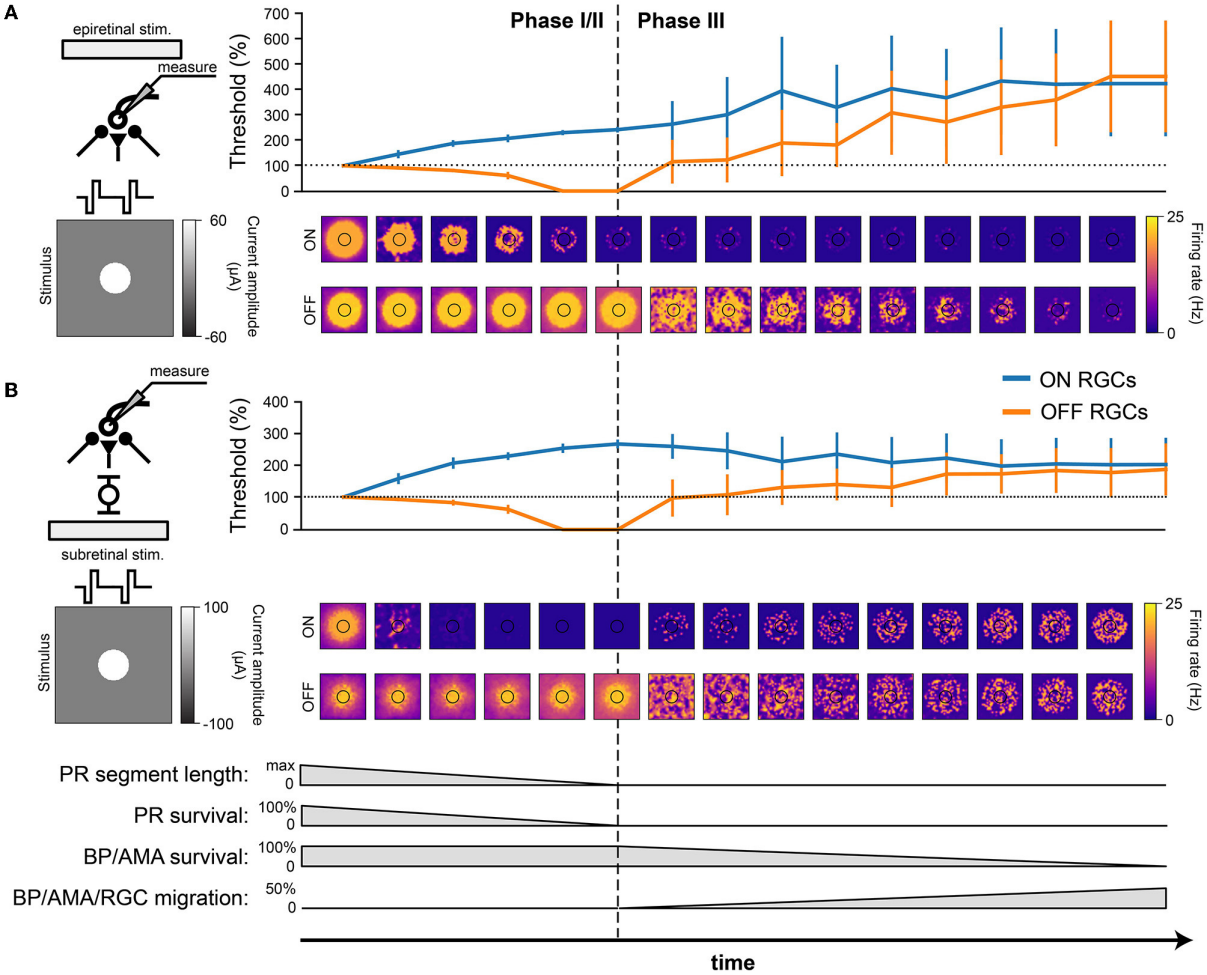
RGC response to electrical stimulation with a 20 Hz biphasic cathodic-pulse train (0.45 ms phase duration, 1 s stimulus duration). Mean values were calculated over 1,000 ms (stimulus presentation) and averaged across neurons located directly below the electrode; vertical bars the SD. **(A)** Epiretinal stimulation. Electrical stimulation thresholds during retinal degeneration reported relative to healthy thresholds (100 % horizontal dotted line). The spatial response profile of ON and OFF RGCs is given below for a pulse train of 60 μA amplitude. The borders of the electrode are outlined in black. **(B)** Subretinal stimulation. Electrical stimulation thresholds during retinal degeneration reported relative to healthy thresholds (100 %, horizontal dotted line). The spatial response profile of ON and OFF RGCs is given below for a pulse train of 60 μA amplitude. The borders of the electrode are outlined in black.

Understanding how the degenerated retina responds to electrical stimulation is crucial for treatment options such as retinal prostheses. We thus placed a simulated disk electrode (80 μm) either epiretinally (i.e., 2 μm above the ganglion cell layer; Cottaris and Elfar, 2005) or subretinally (i.e., at *z* = 135μm, close to horizontal and bipolar cells) and measured the RGC response to a 20 Hz cathodic-first biphasic pulse train of 1 s duration with 0.45 ms phase duration (Figure 7).

Consistent with the literature (Rizzo et al., 2003b; O'Hearn et al., 2006; Jensen and Rizzo, 2008; Goo et al., 2011; Cho et al., 2016), electrical thresholds in later stages of degeneration rose to 200–400% of those in the healthy retina (Figure 7). Here, threshold was defined as the smallest stimulus amplitude that elicited a spike on at least half of 20 trials (Sekirnjak et al., 2009; Weitz et al., 2014), and the resulting thresholds were averaged across the population of surviving RGCs.

Interestingly, our model predicted that degeneration should affect the electrical thresholds of ON and OFF RGCs differently: whereas thresholds for ON cells tended to rise rapidly in Phase I/II, OFF cell thresholds decreased throughout Phase I/II to a point where the threshold was effectively zero, due to increased spontaneous firing. As cells started to migrate in Phase III, thresholds rose again. Notably, epiretinal stimulation thresholds kept rising (Figure 7A), reaching thresholds up to 400 % of those found in a healthy retina, whereas subretinal thresholds were more stable during Phase III and plateaued at around 200 % of the healthy thresholds (Figure 7). The standard deviations in Phase III were significantly larger than those in Phase I/II because of the cell migration in Phase III.

In addition, the spatial response profiles were strongly affected by degeneration (heat maps in Figure 7, here shown for a 20 Hz biphasic pulse train with 60 mA amplitude). A ring-like structure is noticeable in both ON and OFF cell responses due to the influence of the extracellular potential being highest along the edge of the electrode, allowing activity to spread far beyond the size of the electrode. Epiretinal stimulation was able to elicit solid OFF cell responses throughout Phase I/II, whereas ON cell responses lasted only halfway through. After that, cell migration started to disrupt spatial response profiles in Phase III. The story was similar for subretinal stimulation, though a few differences were noticeable. First, ON cell responses vanished almost immediately in Phase I/II, only to come back in late stages of Phase III as more and more ganglion cells started to migrate closer to the subretinal electrode. Second, the spatial activation profile of OFF cells was more confined early in Phase I/II, but began to widen due to increased spontaneous RGC later in Phase I/II. Third, cell migration disrupted the spatial response profiles more quickly and more thoroughly, but continued to elicit responses all the way to the end of Phase III.

### 3.6. Cell death and migration affect ON and OFF cells differently

**Figure 8:**
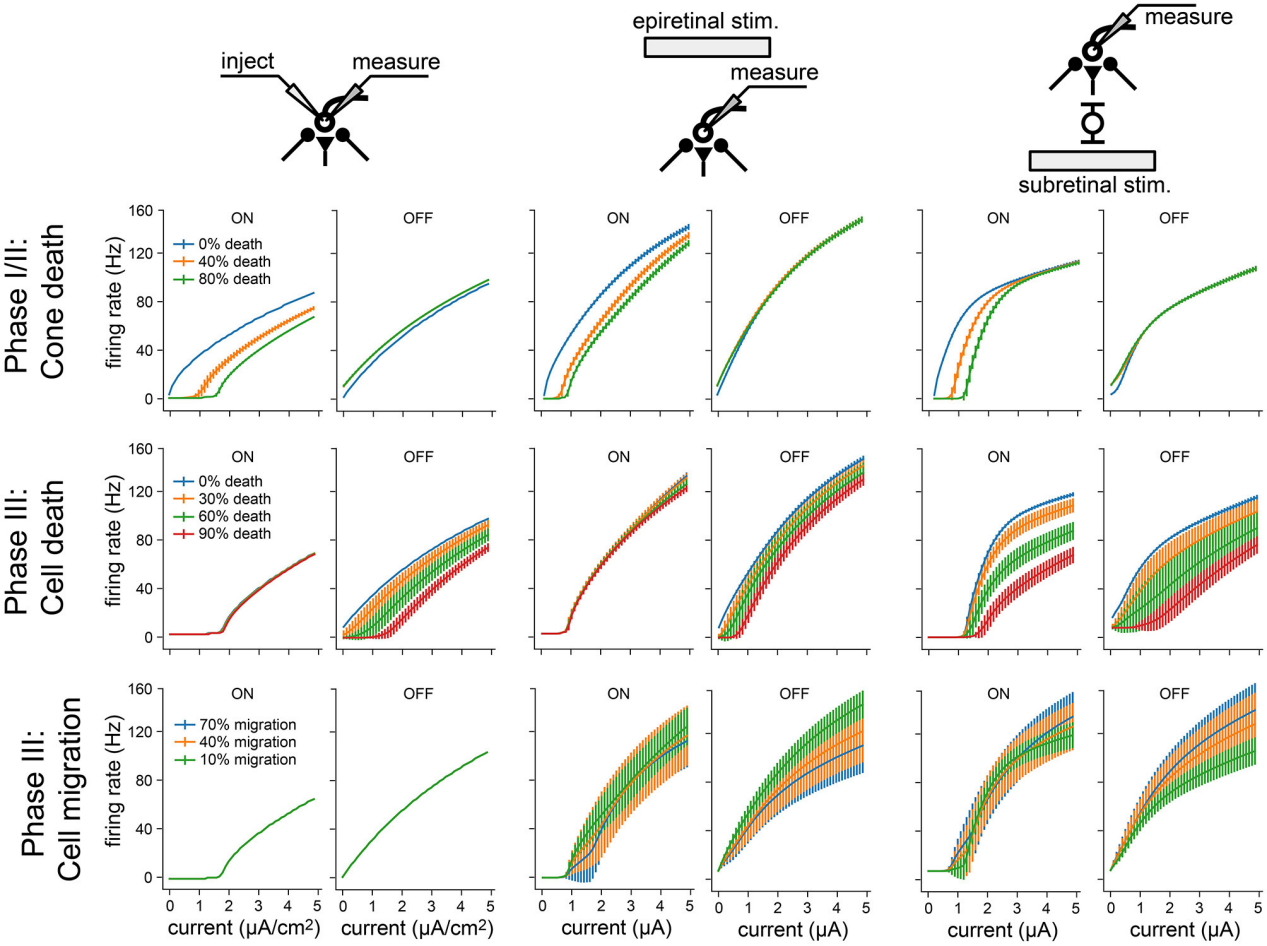
Frequency-current (F-I) curves for three modes of neuronal stimulation: current injection (*two leftmost columns*), epiretinal electrical stimulation (*two center columns*), and subretinal electrical stimulation (*two rightmost columns*). F-I curves are shown for RGCs at different stages of retinal degeneration: as a function of cone death during Phase I/II (top), as a function of bipolar and amacrine cell death during Phase III (middle), and as a function of bipolar, amacrine, and ganglion cell migration during Phase III (bottom). Values averaged across RGCs; vertical bars are the standard deviation.

To isolate the network changes responsible for the altered electrical response properties of RGCs, we simulated frequency-current (F-I) curves at different stages of degeneration for three different stimulation modes: current injection, epiretinal electrical stimulation, and subretinal electrical stimulation (Figure 8). During Phase I/II, cone death reduced the response of ON cells for all three stimulation modes (Figure 8, *top row*), but left OFF cells unaffected. This is consistent with the retina's light response in Figure 4F. During Phase III, bipolar and amacrine cell death reduced the response of OFF cells for all three stimulation modes, but left the ON cells mostly unaffected (Figure 8, *top row*); the one exception being subretinally stimulated ON cells, which saw the greatest reduction in activity. Finally, cell migration affected the response of both ON and OFF cells for both epiretinal and subretinal stimulation, increasing response variability across the RGC population.

Overall, these results demonstrate how network-level changes may affect RGC firing at different stages of retinal degeneration.

## 4. Discussion

We have developed a biophysically inspired *in silico* model of the cone pathway in the retina that simulates the network-level response to both light and electrical stimulation, and found that simulated cone-mediated retinal degeneration differentially affects ON and OFF RGCs. Existing computational models of retinal degeneration largely focus on RGC activity in the absence of photoreceptor input (e.g., Cottaris and Elfar, 2005; Golden et al., 2018), but do not consider the global retinal remodeling that may impact the responsiveness of RGCs. To this end, our simulations do not just reproduce commonly reported findings about the changes in RGC activity encountered during retinal degeneration (e.g., hyperactivity, increased electrical thresholds) but also offer testable predictions about the neuroanatomical mechanisms that may underlie altered RGC activity as a function of disease progression.

Consistent with the literature (Pu et al., 2006; Stasheff, 2008; Sekirnjak et al., 2011; Stasheff et al., 2011; Telias et al., 2019), we found that RGCs exhibited elevated spontaneous firing rates during retinal degeneration (Figure 4). In our model this was mainly restricted to the OFF RGC population, which became more active over time (Figures 4D, G). Similar observations have been made in degenerated retinas of mouse models (Pu et al., 2006; Stasheff, 2008; Sekirnjak et al., 2011), which are dominated by OFF cell activity. However, we identified photoreceptor cell death in Phase I/II as the main driving force behind this hyperactivity, as opposed to an intrinsic change to RGC excitability (Telias et al., 2019). Moreover, the complete loss of cones did not drive RGCs into an oscillatory state (Figure A2). However, this may be due to the fact that our simulations did not include gap junctions (see below). On the other hand, ON cells showed increased activity in response to cone outer segment truncation (Figure 4F), which may occur only early in degeneration before most photoreceptors are lost, after which their spontaneous activity is expected to quickly vanish.

As the light response of the RGC population slowly subsided (Figure 5), ON cells saw a much quicker reduction in firing rate than OFF cells, remaining silent for the second half of Phase I/II and all throughout Phase III (Figure 5). A generalized linear model revealed a brief broadening of the spatiotemporal receptive field for ON RGCs early in Phase I/II while these cells were losing their inhibitory surround, before the spatial properties of both ON and OFF receptive field were lost (Figure 6). This is consistent with studies that have documented cell type–specific functional changes in RGCs across animal models (Pu et al., 2006; Sekirnjak et al., 2011; Yu et al., 2017), where spatial receptive fields often lose their circular shape and appear spotty before they vanish (Yu et al., 2017).

As degeneration progressed, electrical thresholds tended to increase for both subretinal and epiretinal stimulation (Figure 8), which is consistent with most literature on the subject (Rizzo et al., 2003b; O'Hearn et al., 2006; Jensen and Rizzo, 2008; Goo et al., 2011; Cho et al., 2016)—though see Sekirnjak et al. (2009). Mirroring the changes in the light response, ON cells also displayed diminished responses to electrical stimulation (Figure 8). This resulted in higher activation thresholds for ON cells as compared to OFF cells (Figure 7), which is a phenomenon previously documented in the degenerated mouse retina (Yang et al., 2018). Interestingly, our model also predicts a brief period of degeneration during which OFF cells are so active that their electrical threshold is effectively zero. Furthermore, our model offers testable predictions of how cone death (Phase I/II), bipolar and amacrine cell death (Phase III), and cell migration (Phase III) affect the responsiveness of RGCs (Figure 8).

Overall, our findings demonstrate how biophysical changes associated with retinal degeneration affect retinal responses to both light and electrical stimulation, which may have important implications for the design and application of retinal prostheses. In specific, our results suggest that spatially confined responses might be more easily elicited with subretinal stimulation in Phase I/II and with epiretinal stimulation in Phase III (Figure 7). This implies a role for subretinal prostheses in early stages of the disease, whereas epiretinal prostheses may be more effective in later stages. However, cell migration might strongly affect both stimulation modes (Figure 8), leading to spotty activation of the RGC population that may obscure the perceptual interpretation of these electrical stimuli.

Moreover, both subretinal and epiretinal stimulation are expected to more easily activate OFF cells, as they remain active through most of Phase III. This suggests that OFF cell activity may play a greater role in prosthetic vision than previously assumed (Im and Fried, 2015), which could have important implications for the differential activation of RGC subtypes (Twyford et al., 2014; Yang et al., 2018; Chang et al., 2019; Tong et al., 2020).

Although our model captures a range of biophysical changes common to cone-mediated retinal degeneration, we were forced to make some simplifying assumptions due to the complex nature of the degeneration process. Most notably, our model did not include rod circuitry and gap junctions. This may explain why we did not observe oscillations in our model, since rod bipolar cells are thought to participate in an oscillatory network in the outer retina (Haq et al., 2014; Euler and Schubert, 2015) that may arise from electrically coupled networks (Yee et al., 2012; Haq et al., 2014; Euler and Schubert, 2015; Trenholm and Awatramani, 2015; Goo et al., 2016; Ahn et al., 2022). In addition, we did not consider it feasible to model the hyperproliferation of Muller cells, the formation of microneuromas, or other morphological changes commonly observed during inherited retinal degeneration (Marc et al., 2003).

Nevertheless, as we did not modify the intrinsic properties of the RGC population, our results suggest that commonly documented physiological changes such as RGC hyperactivity and increased electrical thresholds (Chen et al., 2013; Saha et al., 2016; Telias et al., 2019) may have additional network-mediate causes that are presynaptic to RGCs. This work thus offers testable predictions to further our understanding of retinal processing in health and disease. Future work could focus on adding additional morphological and topological detail to the simulation in order to obtain a more complete picture of the changes in RGC response properties associated with inherited retinal degeneration.

## Data availability statement

The original contributions presented in the study are included in the article/Supplementary material, further inquiries can be directed to the corresponding author/s.

## Author contributions

AX and MB designed the study and wrote the paper. AX implemented the simulations. All authors contributed to the article and approved the submitted version.

## References

[B1] Ahn J. Cha S. Choi K.-E. Kim S.-W. Yoo Y. Goo Y. S. (2022). Correlated activity in the degenerate retina inhibits focal response to electrical stimulation. Front. Cell. Neurosci. 16:889663. 10.3389/fncel.2022.889663 35602554 PMC9114441

[B2] Beyeler M. (2019). “Biophysical model of axonal stimulation in epiretinal visual prostheses,” in 2019 9th International IEEE/EMBS Conference on Neural Engineering (NER) (San Francisco, CA), 348–351. 10.1109/NER.2019.8716969

[B3] Beyeler M. Nanduri D. Weiland J. D. Rokem A. Boynton G. M. Fine I. (2019). A model of ganglion axon pathways accounts for percepts elicited by retinal implants. Sci. Rep. 9, 1–16. 10.1038/s41598-019-45416-4 31235711 PMC6591412

[B4] Chang Y.-C. Ghaffari D. H. Chow R. H. Weiland J. D. (2019). Stimulation strategies for selective activation of retinal ganglion cell soma and threshold reduction. J. Neural Eng. 16:026017. 10.1088/1741-2552/aaf92b 30560810 PMC6648650

[B5] Chen Z. Song Y. Yao J. Weng C. Yin Z. Q. (2013). Alterations of sodium and potassium channels of RGCs in RCS rat with the development of retinal degeneration. J. Mol. Neurosci. 51, 976–985. 10.1007/s12031-013-0082-9 23934450

[B6] Cho A. Ratliff C. Sampath A. Weiland J. (2016). Changes in ganglion cell physiology during retinal degeneration influence excitability by prosthetic electrodes. J. Neural Eng. 13:025001. 10.1088/1741-2560/13/2/025001 26905177 PMC4852154

[B7] Cottaris N. P. Elfar S. D. (2005). How the retinal network reacts to epiretinal stimulation to form the prosthetic visual input to the cortex. J. Neural Eng. 2, S74–S90. 10.1088/1741-2560/2/1/010 15876658

[B8] Crooks J. Kolb H. (1992). Localization of GABA, glycine, glutamate and tyrosine hydroxylase in the human retina. J. Compar. Neurol. 315, 287–302. 10.1002/cne.903150305 1346792

[B9] Euler T. Schubert T. (2015). Multiple independent oscillatory networks in the degenerating retina. Front. Cell. Neurosci. 9:444. 10.3389/fncel.2015.00444 26617491 PMC4637421

[B10] Fohlmeister J. Coleman P. Miller R. (1990). Modeling the repetitive firing of retinal ganglion cells. Brain Res. 510, 343–345. 10.1016/0006-8993(90)91388-W 2331606

[B11] Fohlmeister J. F. Miller R. F. (1997). Impulse encoding mechanisms of ganglion cells in the tiger salamander retina. J. Neurophysiol. 78, 1935–1947. 10.1152/jn.1997.78.4.1935 9325362

[B12] Fried S. I. Lasker A. C. W. Desai N. J. Eddington D. K. Rizzo J. F. (2009). Axonal sodium-channel bands shape the response to electric stimulation in retinal ganglion cells. J. Neurophysiol. 101, 1972–1987. 10.1152/jn.91081.2008 19193771 PMC4588392

[B13] Golden J. R. Erickson-Davis C. Cottaris N. P. Parthasarathy N. Rieke F. Brainard D.. (2018). Simulation of visual perception and learning with a retinal prosthesis. J. Neural Eng. 10.1101/206409 30523985

[B14] Goo Y. S. Park D. J. Ahn J. R. Senok S. S. (2016). Spontaneous oscillatory rhythms in the degenerating mouse retina modulate retinal ganglion cell responses to electrical stimulation. Front. Cell. Neurosci. 9:512. 10.3389/fncel.2015.00512 26793063 PMC4709854

[B15] Goo Y. S. Ye J. H. Lee S. Nam Y. Ryu S. B. Kim K. H. (2011). Retinal ganglion cell responses to voltage and current stimulation in wild-type and rd1 mouse retinas. J. Neural Eng. 8:035003. 10.1088/1741-2560/8/3/035003 21593549

[B16] Greenberg R. J. Velte T. J. Humayun M. S. Scarlatis G. N. de Juan E. (1999). A computational model of electrical stimulation of the retinal ganglion cell. IEEE Trans. Biomed. Eng. 46, 505–514. 10.1109/10.759051 10230129

[B17] Guo T. Tsai D. Bai S. Morley J. W. Suaning G. J. Lovell N. H.. (2014). Understanding the retina: a review of computational models of the retina from the single cell to the network level. Crit. Rev. Biomed. Eng. 42, 419–436. 10.1615/CritRevBiomedEng.2014011732 25745804

[B18] Guo T. Tsai D. Morley J. W. Suaning G. J. Kameneva T. Lovell N. H.. (2016). Electrical activity of ON and OFF retinal ganglion cells: a modelling study. J. Neural Eng. 13:025005. 10.1088/1741-2560/13/2/025005 26905646

[B19] Haq W. Arango-Gonzalez B. Zrenner E. Euler T. Schubert T. (2014). Synaptic remodeling generates synchronous oscillations in the degenerated outer mouse retina. Front. Neural Circuits 8:108. 10.3389/fncir.2014.00108 25249942 PMC4155782

[B20] Im M. Fried S. I. (2015). Indirect activation elicits strong correlations between light and electrical responses in ON but not OFF retinal ganglion cells. J. Physiol. 593, 3577–3596. 10.1113/JP270606 26033477 PMC4560585

[B21] Jensen R. J. Rizzo J. F. (2008). Activation of retinal ganglion cells in wild-type and rd1 mice through electrical stimulation of the retinal neural network. Vis. Res. 48, 1562–1568. 10.1016/j.visres.2008.04.016 18555890

[B22] Jones B. W. Pfeiffer R. L. Ferrell W. D. Watt C. B. Marmor M. Marc R. E. (2016). Retinal remodeling in human retinitis pigmentosa. Exp. Eye Res. 150, 149–165. 10.1016/j.exer.2016.03.018 27020758 PMC5031517

[B23] Knuth D. (1969). The Art of Computer Programming: Volume 2: Seminumerical Algorithms. Addison-Wesley Series in Computer Science & Information Processing. Reading [etc.]. Boston, MA: Addison-Wesley Publishing Company.

[B24] Kolb H. Nelson R. F. Ahnelt P. K. Ortuño-Lizarán I. Cuenca N. (1995). “The architecture of the human fovea,” in Webvision: The Organization of the Retina and Visual System, eds H. Kolb, E. Fernandez, and R. Nelson (Salt Lake City, UT: University of Utah Health Sciences Center). 32129967

[B25] Loizos K. Marc R. Humayun M. Anderson J. R. Jones B. W. Lazzi G. (2018). Increasing electrical stimulation efficacy in degenerated retina: stimulus waveform design in a multiscale computational model. IEEE Trans. Neural Syst. Rehabil. Eng. 26, 1111–1120. 10.1109/TNSRE.2018.2832055 29877835 PMC6005361

[B26] Luo Y. H.-L. Zhong J. J. Clemo M. Cruz L. d. (2016). Long-term repeatability and reproducibility of phosphene characteristics in chronically implanted Argus II retinal prosthesis subjects. Am. J. Ophthalmol. 170, 100–109. 10.1016/j.ajo.2016.07.021 27491695

[B27] Marc R. E. Jones B. W. Watt C. B. Strettoi E. (2003). Neural remodeling in retinal degeneration. Prog. Retinal Eye Res. 22, 607–655. 10.1016/S1350-9462(03)00039-9 12892644

[B28] Margolis D. J. Gartland A. J. Singer J. H. Detwiler P. B. (2014). Network oscillations drive correlated spiking of ON and OFF ganglion cells in the rd1 mouse model of retinal degeneration. PLoS ONE 9:e86253. 10.1371/journal.pone.0086253 24489706 PMC3904909

[B29] McIntosh L. Maheswaranathan N. Nayebi A. Ganguli S. Baccus S. (2016). “Deep learning models of the retinal response to natural scenes,” in Advances in Neural Information Processing Systems 29, eds D. D. Lee, M. Sugiyama, U. V. Luxburg, I. Guyon, and R. Garnett (Barcelona: Curran Associates, Inc.), 1369–1377. PMC5515384 28729779

[B30] Milo R. Jorgensen P. Moran U. Weber G. Springer M. (2010). BioNumbers-the database of key numbers in molecular and cell biology. Nucl. Acids Res. 38, D750–D753. 10.1093/nar/gkp889 19854939 PMC2808940

[B31] O'Hearn T. M. Sadda S. R. Weiland J. D. Maia M. Margalit E. Humayun M. S. (2006). Electrical stimulation in normal and retinal degeneration (rd1) isolated mouse retina. Vis. Res. 46, 3198–3204. 10.1016/j.visres.2006.03.031 16723150

[B32] Paknahad J. Loizos K. Humayun M. Lazzi G. (2020). Targeted stimulation of retinal ganglion cells in epiretinal prostheses: a multiscale computational study. IEEE Trans. Neural Syst. Rehabil. Eng. 28, 2548–2556. 10.1109/TNSRE.2020.3027560 32991284 PMC7737501

[B33] Paknahad J. Loizos K. Yue L. Humayun M. S. Lazzi G. (2021). Color and cellular selectivity of retinal ganglion cell subtypes through frequency modulation of electrical stimulation. Sci. Rep. 11, 1–13. 10.1038/s41598-021-92050-0 33664347 PMC7933163

[B34] Palanker D. Le Mer Y. Mohand-Said S. Muqit M. Sahel J. A. (2020). Photovoltaic restoration of central vision in atrophic age-related macular degeneration. Ophthalmology 127, 1097–1104. 10.1016/j.ophtha.2020.02.024 32249038 PMC7384969

[B35] Pu M. Xu L. Zhang H. (2006). Visual response properties of retinal ganglion cells in the royal college of surgeons dystrophic rat. Invest. Ophthalmol. Vis. Sci. 47, 3579–3585. 10.1167/iovs.05-1450 16877432

[B36] Rizzo J. F. Wyatt J. Loewenstein J. Kelly S. Shire D. (2003a). Perceptual efficacy of electrical stimulation of human retina with a microelectrode array during short-term surgical trials. Invest. Ophthalmol. Vis. Sci. 44, 5362–5369. 10.1167/iovs.02-0817 14638739

[B37] Rizzo J. F. III. Wyatt J. Loewenstein J. Kelly S. Shire D. (2003b). Methods and perceptual thresholds for short-term electrical stimulation of human retina with microelectrode arrays. Invest. Ophthalmol. Vis. Sci. 44, 5355–5361. 10.1167/iovs.02-0819 14638738

[B38] Rodieck R. W. (1998). The First Steps in Seeing. Sunderland, MA: Sinauer Associates.

[B39] Saha S. Greferath U. Vessey K. A. Grayden D. B. Burkitt A. N. Fletcher E. L. (2016). Changes in ganglion cells during retinal degeneration. Neuroscience 329, 1–11. 10.1016/j.neuroscience.2016.04.032 27132232

[B40] Sekirnjak C. Hulse C. Jepson L. H. Hottowy P. Sher A. Dabrowski W.. (2009). Loss of responses to visual but not electrical stimulation in ganglion cells of rats with severe photoreceptor degeneration. J. Neurophysiol. 102, 3260–3269. 10.1152/jn.00663.2009 19726725 PMC2804428

[B41] Sekirnjak C. Jepson L. H. Hottowy P. Sher A. Dabrowski W. Litke A. M.. (2011). Changes in physiological properties of rat ganglion cells during retinal degeneration. J. Neurophysiol. 105, 2560–2571. 10.1152/jn.01061.2010 21389304 PMC3094174

[B42] Shi Q. Gupta P. Boukhvalova A. K. Singer J. H. Butts D. A. (2019). Functional characterization of retinal ganglion cells using tailored nonlinear modeling. Sci. Rep. 9:8713. 10.1038/s41598-019-45048-8 31213620 PMC6581951

[B43] Smith R. G. (1995). Simulation of an anatomically defined local circuit: the cone-horizontal cell network in cat retina. Vis. Neurosci. 12, 545–561. 10.1017/S0952523800008440 7654610

[B44] Stasheff S. F. (2008). Emergence of sustained spontaneous hyperactivity and temporary preservation of off responses in ganglion cells of the retinal degeneration (rd1) mouse. J. Neurophysiol. 99, 1408–1421. 10.1152/jn.00144.2007 18216234

[B45] Stasheff S. F. Shankar M. Andrews M. P. (2011). Developmental time course distinguishes changes in spontaneous and light-evoked retinal ganglion cell activity in rd1 and rd10 mice. J. Neurophysiol. 105, 3002–3009. 10.1152/jn.00704.2010 21389300

[B46] Stimberg M. Brette R. Goodman D. F. (2019). Brian 2, an intuitive and efficient neural simulator. eLife 8:e47314. 10.7554/eLife.47314 31429824 PMC6786860

[B47] Stimberg M. Goodman D. F. M. Nowotny T. (2020). Brian2GeNN: accelerating spiking neural network simulations with graphics hardware. Sci. Rep. 10:410. 10.1038/s41598-019-54957-7 31941893 PMC6962409

[B48] Stingl K. Bartz-Schmidt K. U. Besch D. Braun A. Bruckmann A. Gekeler F.. (2013). Artificial vision with wirelessly powered subretinal electronic implant alpha-IMS. Proc. Biol. Sci. 280:20130077. 10.1098/rspb.2013.0077 23427175 PMC3619489

[B49] Tao X. Sabharwal J. Wu S. M. Frankfort B. J. (2020). Intraocular pressure elevation compromises retinal ganglion cell light adaptation. Invest. Opthalmol. Vis. Sci. 61:15. 10.1167/iovs.61.12.15 33064129 PMC7571289

[B50] Telias M. Denlinger B. Helft Z. Thornton C. Beckwith-Cohen B. Kramer R. H. (2019). Retinoic acid induces hyperactivity, and blocking its receptor unmasks light responses and augments vision in retinal degeneration. Neuron 102, 574–586.e5. 10.1016/j.neuron.2019.02.015 30876849 PMC6508985

[B51] Tong W. Meffin H. Garrett D. J. Ibbotson M. R. (2020). stimulation strategies for improving the resolution of retinal prostheses. Front. Neurosci. 14:262. 10.3389/fnins.2020.00262 32292328 PMC7135883

[B52] Trenholm S. Awatramani G. B. (2015). Origins of spontaneous activity in the degenerating retina. Front. Cell. Neurosci. 9:277. 10.3389/fncel.2015.00277 26283914 PMC4518194

[B53] Twyford P. Cai C. Fried S. (2014). Differential responses to high-frequency electrical stimulation in ON and OFF retinal ganglion cells. J. Neural Eng. 11:025001. 10.1088/1741-2560/11/2/025001 24556536 PMC4465532

[B54] Wassle H. Boycott B. B. (1991). Functional architecture of the mammalian retina. Physiol. Rev. 71, 447–480. 10.1152/physrev.1991.71.2.447 2006220

[B55] Weitz A. C. Behrend M. R. Ahuja A. K. Christopher P. Wei J. Wuyyuru V.. (2014). Interphase gap as a means to reduce electrical stimulation thresholds for epiretinal prostheses. J. Neural Eng. 11:016007. 10.1088/1741-2560/11/1/016007 24654269 PMC3970787

[B56] Werginz P. Im M. Hadjinicolaou A. E. Fried S. I. (2018). “Visual and electric spiking responses of seven types of rabbit retinal ganglion cells,” in 2018 40th Annual International Conference of the IEEE Engineering in Medicine and Biology Society (EMBC) (Honolulu, HI), 2434–2437. 10.1109/EMBC.2018.8512746 30440899

[B57] Wiley J. D. Webster J. G. (1982). Analysis and control of the current distribution under circular dispersive electrodes. IEEE Trans. Biomed. Eng. 29, 381–385. 10.1109/TBME.1982.324910 7084970

[B58] Wohrer A. Kornprobst P. (2009). Virtual retina: a biological retina model and simulator, with contrast gain control. J. Comput. Neurosci. 26, 219–249. 10.1007/s10827-008-0108-4 18670870

[B59] Wyatt J. Rizzo J. (1996). Ocular implants for the blind. IEEE Spectr. 33, 47–53. 10.1109/6.490056

[B60] Yang C. Y. Tsai D. Guo T. Dokos S. Suaning G. J. Morley J. W.. (2018). Differential electrical responses in retinal ganglion cell subtypes: effects of synaptic blockade and stimulating electrode location. J. Neural Eng. 15:046020. 10.1088/1741-2552/aac315 29737971

[B61] Yee C. W. Toychiev A. H. Sagdullaev B. T. (2012). Network deficiency exacerbates impairment in a mouse model of retinal degeneration. Front. Syst. Neurosci. 6:8. 10.3389/fnsys.2012.00008 22383900 PMC3285818

[B62] Yu W.-Q. Grzywacz N. M. Lee E.-J. Field G. D. (2017). Cell type-specific changes in retinal ganglion cell function induced by rod death and cone reorganization in rats. J. Neurophysiol. 118, 434–454. 10.1152/jn.00826.2016 28424296 PMC5506261

[B63] Zeck G. Lambacher A. Fromherz P. (2011). Axonal transmission in the retina introduces a small dispersion of relative timing in the ganglion cell population response. PLoS ONE 6:e20810. 10.1371/journal.pone.0020810 21674067 PMC3107248

