# Supplementary material for: Retinal ganglion cells undergo cell type—specific functional changes in a computational model of cone-mediated retinal degeneration
Source: Front Neurosci. 2023 May 18;17:1147729. doi: 10.3389/fnins.2023.1147729 (PMC10233015; doi:10.3389/fnins.2023.1147729)
Supplement: Supplementary file 2 [file Data_Sheet_1.PDF]

Appendix A. Supplemental Information

A.1. *Inter-spike intervals*

Fig. A1A shows the inter-spike interval distribution for ON and OFF retinal ganglion cells (RGCs) as a function of disease progression. Fig. A1B highlights the histograms of the inter-spike interval distributions presented in Panel A during Phase I/II. Note that both the mean firing rate (see Fig. 4) and the mean inter-spike interval of ON cells (Fig. A1B, *left*) decreased as degeneration progressed. This is because most ON cells did not spike at all and therefore did not contribute any finite inter-spike interval values. However, a select few did spike a lot, therefore contributing many short inter-spike interval values. As a result, mean inter-spike interval decreased while mean firing rate decreased as well. Furthermore, we did not observe any bursting behavior in the spontaneous firing of RGCs.

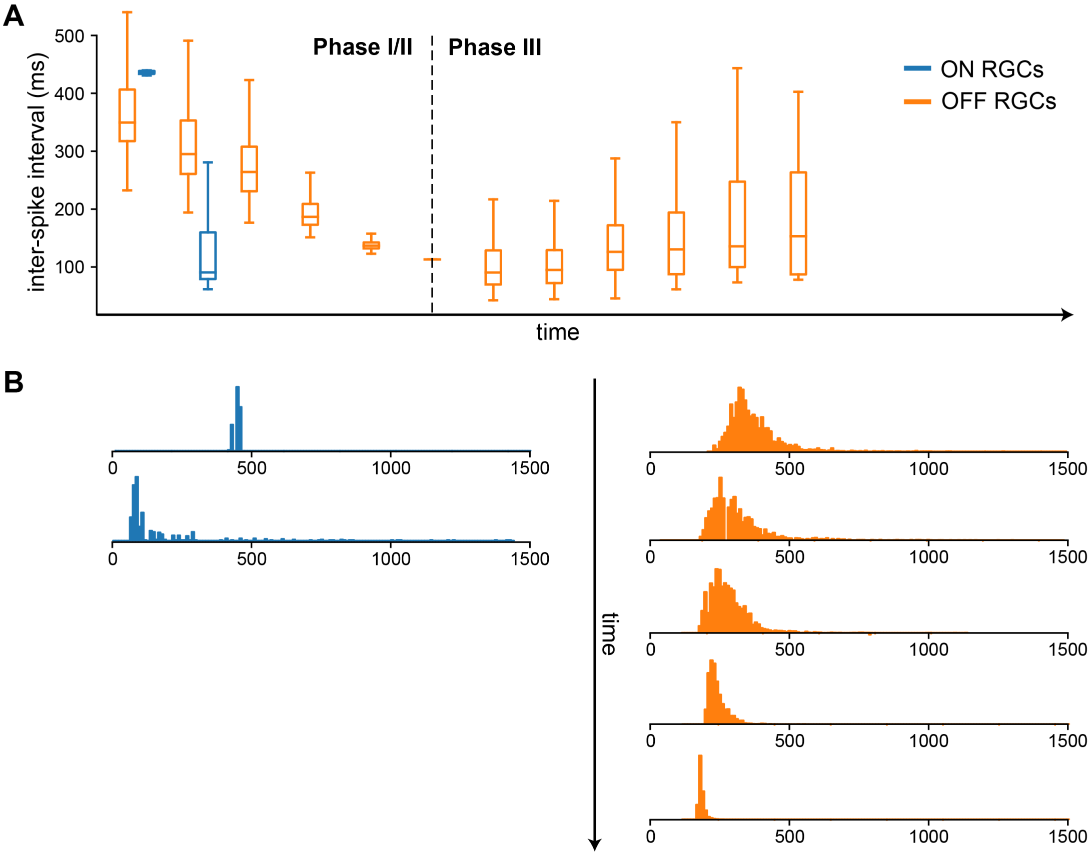

Figure A1. Inter-spike interval distributions as a function of disease progression. **A)** Box plots of inter-spike interval during Phase I/II and Phase III. **B)** Histograms for the data from Phase I/II that is presented in Panel A.

A.2. *Phase I/II: Light response*

Fig. A2 shows the firing rate of ON and OFF retinal ganglion cells (RGCs) in response to full-field stimuli of a given light intensity $l(t) = \text{const.}$ (Eq. 5), averaged across the population of surviving cells, as a function of both cone outer segment truncation (simulated by a reduction in G_{light} and cone survival rate. As light intensity varied

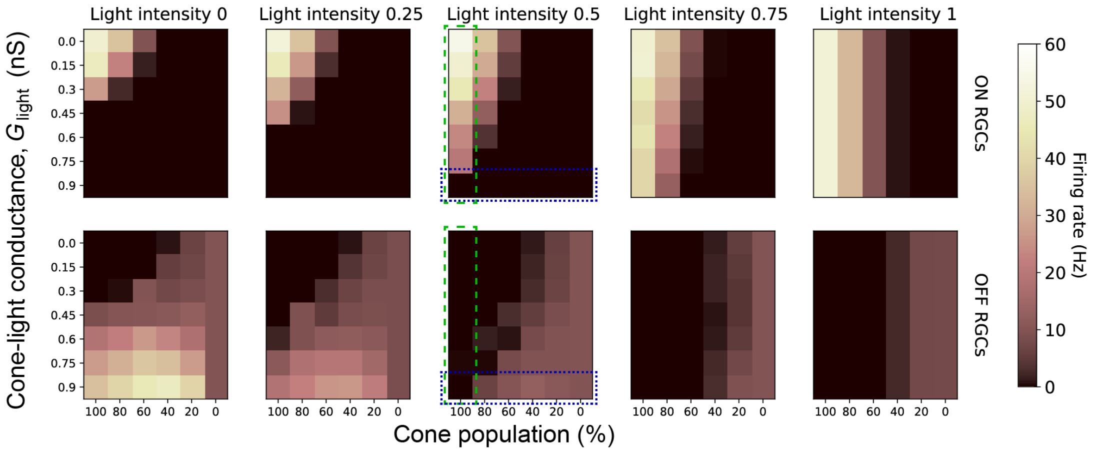

Figure A2. Light response of RGCs as a function of cone outer segment truncation and cone survival rate. The values encapsulated by the green dashed box are the same as the mean values presented in Fig. 4F. The values encapsulated by the blue dotted box are the same as the mean values presented in Fig. 4G.

between 0 (black) and 1 (white), the RGC response to 0.5 intensity was interpreted as the spontaneous firing rate.

At high light intensities ($l(t) > 0.5$), both ON and OFF RGC activity was mainly a function of the size of the surviving cone population. At low light intensities ($l(t) \leq 0.5$), both cone outer segment truncation and cone population size had an effect on RGC firing, with ON firing dominated by outer segment truncation and OFF firing dominated by cone population. The linear time axis of disease progression corresponded to the diagonal in each heatmap (top-left corner: healthy, bottom-right corner: end of Phase I/II).

A.3. *Ganglion cell response to electrical stimulation*

Fig. A3 shows the RGC response to a constant electrical stimulus delivered either epiretinally (Fig. A3A) or subretinally (Fig. A3). The stimulus was the same as for the spatial profiles in Fig. 7; that is, a 20 Hz cathodic-first biphasic pulse train of 1 s duration, with pulse duration 0.45 ms and 60 µA amplitude. Electrode dimesnions are given in Section 2.5.

A.4. *Initial values*

Table A1 shows the initial values for the gating variables in the Hodgkin-Huxley model of the RGCs (Eq. 7). The gating variables are in units of Hz. The internal calcium concentration are in units of nM (nanomolar).

m	h	c	n	A	h_A	y	m_T	h_T	d_T	$[\mathrm{Ca}^{2+}]_i$
0.5	0.5	0.5	0.5	0	1	0.5	0	1	1	0.0001

Table A1. Initial values for the Hodgkin-Huxley model.

Table A2 shows the initial values of the membrane potential for the different cell types. Initial values for each cell type followed a normal distribution with a mean μ and a standard deviation σ.

Cell type	μ (mV)	σ (mV)
cone photoreceptors	−46.8	3
horizontal cells	−47.7	3
ON bipolar cells	−35	3
OFF bipolar cells	−44	3
ON wide-field amacrine cells	−42	3
OFF wide-field amacrine cells	−34.5	3
ON narrow-field amacrine cells	−47.6	3
ON RGCs	−66.5	3
OFF RGCs	−70.5	3

Table A2. Initial values for the membrane potential of the different cell types.

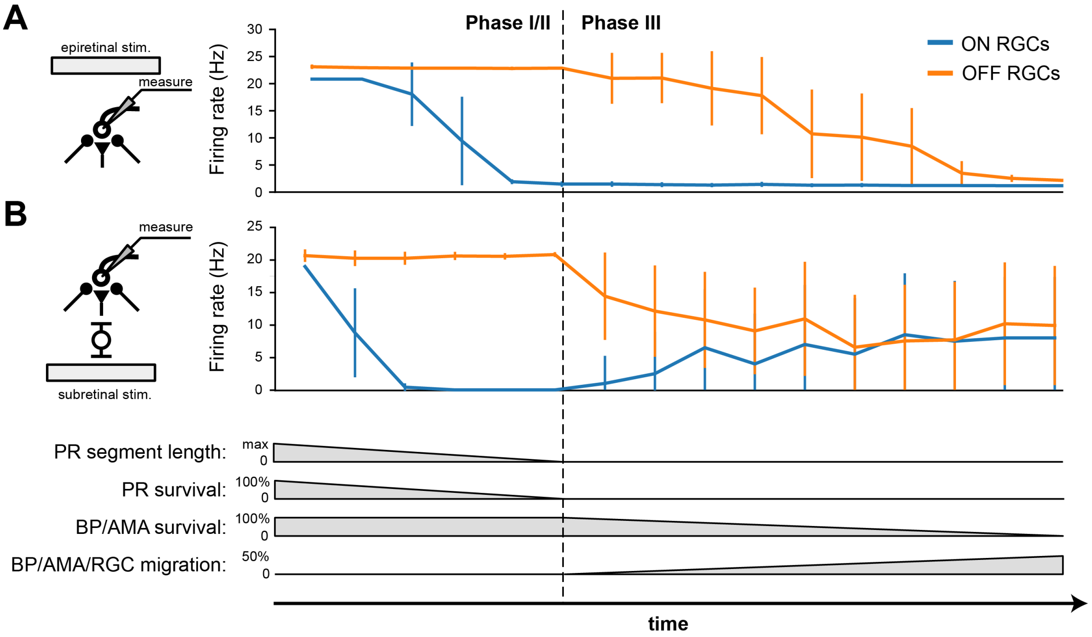

Figure A3. RGC firing rate in response to epiretinal (A) and subretinal (B) stimulation. The mean and standard deviation were calculated from the ON and OFF RGCs that were located directly under the electrode.
